# Research on active collision avoidance control technology for intelligent connected monorail transit trains in the virtual coupling environment

**DOI:** 10.1371/journal.pone.0342193

**Published:** 2026-03-10

**Authors:** Zhongwei Hou, Han Liang, Guang Yang, Jin Han

**Affiliations:** 1 Institute of Future Civil Engineering Science and Technology, Chongqing Jiaotong University, Chongqing, China; 2 Institute of Frontier Interdisciplinary Technology, Chongqing Jiaotong University, Chongqing, China; 3 School of Information Science and Engineering, Chongqing Jiaotong University, Chongqing, China; Southwest Jiaotong University, CHINA

## Abstract

The development of an intelligent connected monorail transit system offers an effective solution to the mismatch between passenger flow and system capacity at various time intervals within urban rail networks. As the core of such a system lies the virtual coupling (VC) technology, which dynamically adjusts train configurations in response to real-time passenger demand, thereby improving resource utilization. However, during VC operations, severe communication delays between vehicles or the sudden emergence of obstacles ahead may still result in rear-end collisions among coupled vehicles, posing significant safety risks. To address these challenges, this paper focuses on the active collision avoidance control of intelligent connected monorail vehicles operating within the VC environment. At the modeling level, a control model is developed to facilitate VC between leading and following vehicles, and the dynamic characteristics of typical operational scenarios—including station approach coupling, tracking coupling, and departure decoupling—are thoroughly analyzed. Building upon this foundation, the train’s behaviors under collision avoidance during accelerated departures, decelerated arrivals, and unexpected obstacle encounters are further investigated. In terms of control strategy, a Model Predictive Control (MPC) algorithm is introduced to enable efficient coordination and proactive collision avoidance among trains. Ultimately, a simulation platform based on Chongqing Rail Transit Line 3 is established for validating the proposed model and algorithm under representative operating scenarios. The evaluation demonstrates gains in system flexibility and safety and technical foundation for the practical implementation of intelligent rail transit systems.

## 1. Introduction

**Fig 1:**
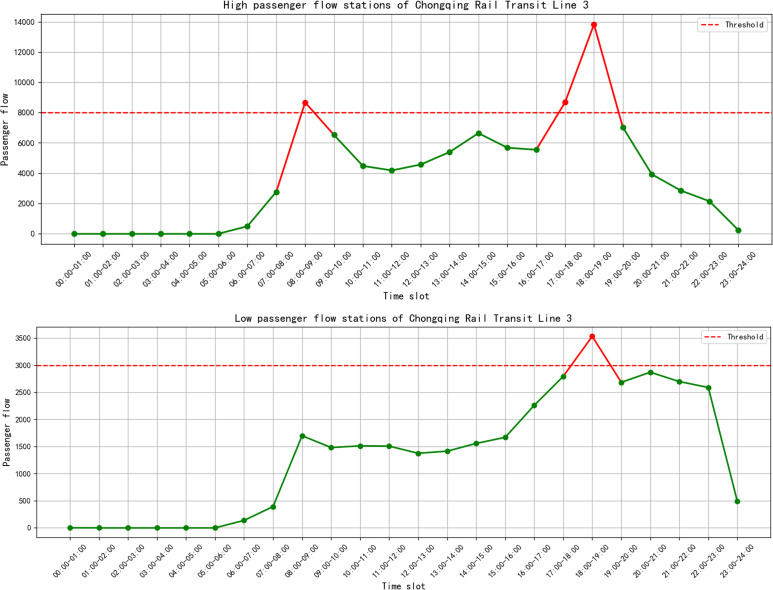
Passenger flow of Chongqing Rail Transit Line 3.

Recent urban expansion has led to the fast growth of rail-based public transport, which now plays a central role in integrated transportation systems. As a sustainable solution to mitigate urban traffic congestion and optimize the allocation of transportation resources, the urban rail transit system has consistently adhered to principles of green, low-carbon, safe, reliable, efficient, and intelligent operation. They not only offer convenient and efficient travel options for urban residents, but also play a vital role in promoting urban modernization and driving economic growth [1–2]. By the end of 2023, a total of 295 urban rail transit lines had been opened across 57 cities in China, with a cumulative operating length of 10,566.55 kilometers. The total passenger volume of these rail transit lines is 175 900 million people. In megacities such as Beijing and Shanghai, the operational length of urban rail networks has exceeded 800 kilometers, with average daily ridership surpassing 10 million passengers [3]. With the rapid development of urban rail transit, the conflict between transit capacity and passenger travel demand has become increasingly prominent. Taking China Chongqing Rail Transit Line 3 as a case study, it is observed that during evening peak hours (17:00–19:00), the maximum hourly load rates at various entry and exit stations exceed 100%, with certain peak stations surpassing 120%. The corresponding distribution of passenger flow is depicted in Fig 1. The existing fixed capacity proves inadequate to accommodate the explosive growth in travel demand. Conversely, during off-peak periods, passenger flow drops significantly, resulting in excess transit capacity.

In-depth analysis indicates that current straddle-type monorail vehicles operate on fixed time intervals and physically coupled multi-car formations. This mode of operation shows a significant mismatch with the dynamic spatiotemporal characteristics of passenger flow, leading to issues such as high operational input and low efficiency. As a result, substantial capacity and vehicle resources are wasted [4]. Against this backdrop, the Intelligent and Connected Monorail Transit System emerges as an integrated and innovative solution of the next-generation rail transit technologies, offering a new technical pathway to address these challenges.

The Intelligent and Connected Monorail Transit System, built upon a monorail infrastructure, integrates advanced technologies such as Vehicle-to-Everything (V2X) communication [5], big data analytics [6], 5G networks [7], and cloud platforms [8]. Internet of Things (IoT)-enabled paradigms have been widely adopted in smart networked systems to enable real-time monitoring, data-driven decision-making, and coordinated optimization, especially when sustainability and resilience are required [9–10]. These developments provide a useful conceptual lens for understanding smart transportation as a cyber–physical system supported by communication, sensing, and computation. This innovative system dynamically configures intelligent vehicle configurations in response to fluctuating passenger demand. Supporting dynamic coupling and decoupling of vehicle units, it significantly enhances both operational safety and transportation efficiency [11]. As a core enabling technology of the system, virtual coupling (VC) allows multiple vehicles to operate collaboratively without physical connections, relying on distributed control and vehicle-to-vehicle (V2V) communication. This enables coordinated movement of multiple vehicle units without mechanical coupling. Compared with a traditional rail transit system characterized by fixed formations and departure intervals, VC utilizes wireless communication to achieve global coordination between leading and following vehicles, thereby reducing headways and enabling high-density, flexible operation. Nevertheless, within the framework of VC, it is crucial to maintain minimal headways between operating Mass Rapid Transit (MRT) vehicle units to maximize line capacity utilization and enhance overall transport capabilities. At this time, if the MRT vehicles are in extreme conditions such as dysfunctional dispatch functions, temporary control system failures, or disruptions in V2V and vehicle-to-infrastructure (V2I) communications, significant safety risks related to potential vehicle collisions persist, posing serious threats to operational safety [12]. However, existing research on VC in rail transit has primarily focused on theoretical analyses [13–14], literature reviews, and simplified models, providing a comprehensive overview of the concept. There is a notable lack of simulation-based studies that incorporate concrete algorithmic models to verify how intelligent and connected MRT vehicles can achieve efficient and reliable collision avoidance under VC scenarios. Current investigations into this subject largely remain confined to traditional collision avoidance strategies, failing to address the specific challenges presented by VC environments.

To address the aforementioned research gap, this study takes the intelligent and connected monorail transit system as the background. Firstly, control models for both leading and following vehicles are developed based on the operational characteristics of vehicle formations under VC. Secondly, to reduce the headway between adjacent MRT vehicles and enhance line capacity, a comprehensive analysis is conducted on various coupling and decoupling scenarios, as well as different collision avoidance strategies, with careful consideration of practical application requirements. Given the relatively straightforward implementation of decoupling, this study primarily concentrates on the coupling phase. Afterward， A Model Predictive Control (MPC) algorithm is employed to achieve both coordinated control during the VC process and proactive collision avoidance under potential hazard scenarios. By following the three core steps of model prediction, rolling optimization, and feedback correction, the MPC framework enables real-time optimization that dynamically adjusts to the current system state [15]. Finally, using the China Chongqing Rail Transit Line 3 as a reference case, the proposed models and algorithms are validated on a dedicated simulation platform. The results demonstrate the feasibility and effectiveness of VC technology in enabling dynamic and efficient vehicle dispatch while ensuring operational safety.

After the introduction, the paper is structured into four main parts. Section 2 summarizes prior work on VC and collision-avoidance control. Section 3 introduces the considered VC operating scenarios and the proposed strategy, including the modeling assumptions and implementation details. Section 4 reports the simulation settings and results, followed by conclusions and future work in Section 5.

## 2. Literature review

### 2.1. Virtual coupling

Traditional train operation control methods divide the track into a series of fixed-length block sections, where only one train is permitted to occupy a block at any given time to ensure a safe separation between trains. The intelligent and connected monorail transit system overcomes the limitations of this conventional approach by employing advanced connectivity to form virtual coupling (VC) through moving block control. The concept of VC was originally applied in conventional railways to reduce the spacing between consecutive trains. This innovation aims to enhance line capacity in response to the rapidly growing demand for rail transportation. Zhang [16] employed a multi-agent approach to model rail transit entities enabled by VC, encompassing train units, train fleets, and representations of passenger attributes and behaviors. Meanwhile, the study proposed mathematical formulations for evaluating train operation costs, passenger travel costs, and riding comfort, validating the effectiveness of the proposed method using the NetLogo simulation platform. Quaglietta et al. [17] introduced the concept of a dynamic safety margin to allow for adaptive adjustment of inter-train spacing under virtual coupling, ensuring the required safety distance was consistently maintained even during hazardous operating scenarios. Wang et al. [18] investigated constraint-force-driven control mechanisms for VC applications. To solve the constraint forces, the Udwadia-Kalaba method was applied to derive explicit equations of motion under equality constraints, and the approach has been validated through simulation. Stanley et al. [19] adopted a PID controller tuned by particle swarm optimization (PSO) for railway trains utilizing VC techniques. By modeling a real-world train together with its corresponding track sections, the superiority of the PSO-tuned PID controller was demonstrated. The success of VC technology in mainline railways has inspired researchers to explore its application in urban rail transit. Chen et al. [20] introduced the fundamental principles and operational characteristics of virtual vehicle formations, proposing organizational modes for urban rail transit based on this concept. Through a comparative analysis of conventional operations and VC-based systems, they evaluated the operational efficiency of urban rail networks and highlighted the advantages of virtual formations through specific case studies. In [21], the existing theoretical studies on vehicle formations operation control are reviewed and categorized into four main approaches: vehicle following, feedback control, optimal control, and computational intelligence methods. The paper also provided sufficient background for understanding the proposed performance indicator (PI) and briefly introduced the operation of VC. Feng et al. [22] conducted a thorough review and analysis of contemporary urban rail transit VC technologies, encompassing the research background, fundamental concepts, and key enabling technologies. Wang et al. [23] proposed an integer linear programming (ILP) model aimed at optimizing train operations on Y-shaped rail lines, encompassing aspects such as train timetables, vehicle circulation plans, and strategies for VC and decoupling. This model allows vehicles to dynamically transition between various configurations during operation. Zheng and Yuan [24] utilized a mixed-integer nonlinear programming (MINLP) model that incorporates virtual formation patterns to optimize vehicle scheduling, rolling stock utilization planning, and passenger flow control strategies. The robustness of this model was validated through real-world data obtained from the Beijing Changping subway line. Zhao et al. [25] developed a multi-objective MILP model and solved a numerical example using CPLEX. A real-world scenario was implemented employing an extended adaptive large neighborhood search (ALNS) algorithm coded in C language. The results indicated that a skip-stop routing strategy integrating VC could substantially reduce total passenger travel time.

### 2.2. Active collision avoidance control

**Fig 2:**
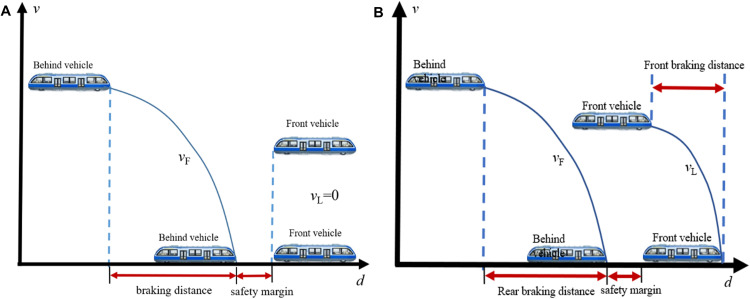
Vehicle braking mode, (A)absolute distance braking mode, ADBM, (B)relative distance braking mode, RDBM.

In moving block signaling systems, MRT vehicles typically follow two standard operational control modes for collision avoidance: Absolute Distance Braking Mode (ADBM) and Relative Distance Braking Mode (RDBM), as illustrated in Fig 2. In ADBM, the braking endpoint is defined as the static tail of the preceding vehicle minus a safety margin, ensuring that the braking distance of the following vehicle remains shorter than the headway distance [26]. This mode is straightforward in principle and possesses a relatively mature technical framework. In contrast, RDBM dynamically calculates a protective speed profile by integrating the preceding vehicle’s real-time position, velocity, and acceleration, thereby enabling continuous and safe following behavior [27]. Due to its enhanced flexibility and improved line throughput, RDBM has become a focal point of research in contemporary intelligent vehicle control systems. Su et al. [28] proposed a novel RDBM framework for safe and efficient following control, which incorporated the predicted trajectory of the preceding vehicle for collision avoidance. They employed a Deep Q-Network (DQN)-based reinforcement learning framework to develop control policies that enabled the following train to achieve both safety and efficiency. Wang et al. [29] developed a vehicle safety control method within the RDBM framework by combining Deep Deterministic Policy Gradient (DDPG) with Control Barrier Function (CBF). Numerical simulations demonstrated that the DDPG-CBF algorithm guarantees safe and stable vehicle operations, resulting in an average improvement in operational efficiency of 38.94%. Zhang et al. [30] explored the integration of spiking neural networks (SNNs) into train control systems. By mimicking the cooperative functioning of different brain regions, SNNs were employed for motion control and planning, effectively preventing overspeed conditions and collisions. Xiao et al. [31] adopted a BP neural network-based PID control approach and conducted simulations using a novel three-dimensional safety braking model based on the dynamic velocities of both leading and following vehicles. Simulation results based on the Beijing Capital Airport Line showed that the safety envelope under the train-to-train communication model improved by over 56.4% compared to conventional models. Chen et al. [32] proposed a distributed global composite learning coordination protocol for heavy-haul train platoons operating under VC. The protocol enables all trains in the platoon to achieve autonomous, dynamically stable coordination with minimum safe spacing while effectively avoiding collisions.

Although existing studies have investigated VC from the perspectives of operational organization, risk modeling, and control design, their focuses remain fragmented, as summarized in Table 1. In particular, there is still a lack of vehicle-level closed-loop validation under typical operational scenarios, as well as proactive collision-avoidance strategies capable of handling emergency or abnormal conditions. Motivated by the above gaps in the literature, the primary contributions of this paper are as follows:

**Table 1:** Classification comparison of research related to virtual coupling (VC).

Category	References	Research focus	Research gap
Operational optimization	[16] [20,23–25]	Improve capacity and efficiency from the system operation perspective	Lack of vehicle-level control and closed-loop collision-avoidance verification
VC mechanism modeling and safety margin analysis	[17] [33–34]	Build VC risk models and analyze dynamic safety margins and risk factors	Mainly focuses on safety analysis and margin modeling; lacks validation of implementable control strategies
VC cooperative control and safety control design	[18,19,26,27,31,32]	Safety control considering uncertainty; optimal/distributed cooperative control	Insufficient coverage of proactive collision avoidance under emergency conditions
Collaborative avoidance and proactive safety protection	[28–30]	Consider collision avoidance under VC or multi-train coordination	Limited engineering-scenario-based and systematic validation

(1) For the first time, to establish a complete VC control model for an intelligent and connected monorail transit system, with a clear distinction between the control strategies of leading and following vehicles.
(2) Conduct a comprehensive analysis of various operational scenarios encountered by MRT vehicles, including coupling, decoupling, and emergency obstacle avoidance, with full consideration of practical application requirements.
(3) Propose and implement an MPC-based algorithm for VC and proactive collision avoidance, and validate its effectiveness and feasibility through simulation experiments.

## 3. Method and principle

### 3.1. Virtual coupling scene analysis

**Fig 3:**
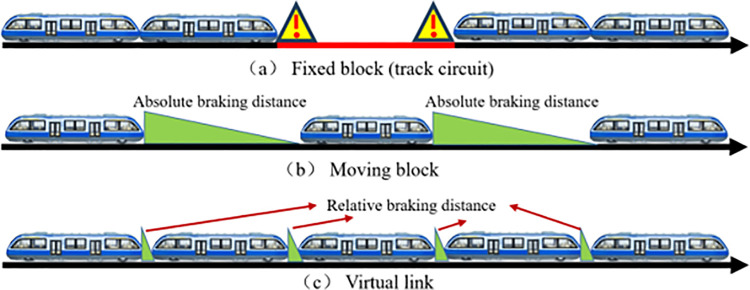
Virtual coupling (VC) model for rail transit.

To dynamically adapt to passenger demand variations across different time periods, the MRT system commonly operates by combining short-formation vehicles into longer formations during peak hours to increase capacity, and by decomposing long formations into shorter ones during off-peak hours to achieve precise matching between supply and demand. Currently, most flexible formation systems still rely on fixed-block or moving-block signaling models to ensure safe separation between vehicles. In contrast, virtual coupling (VC) technology significantly enhances operational efficiency by reducing headways between vehicles and optimizing track resource utilization, thereby supporting fast and flexible vehicle coupling and decoupling. Under the VC mode, MRT vehicles operate within a reduced relative braking distance while synchronizing control commands in real-time, enabling tighter coordination and increased throughput. As illustrated in Fig 3, the VC paradigm offers distinct operational advantages over traditional block-based control models.

**Fig 4:**
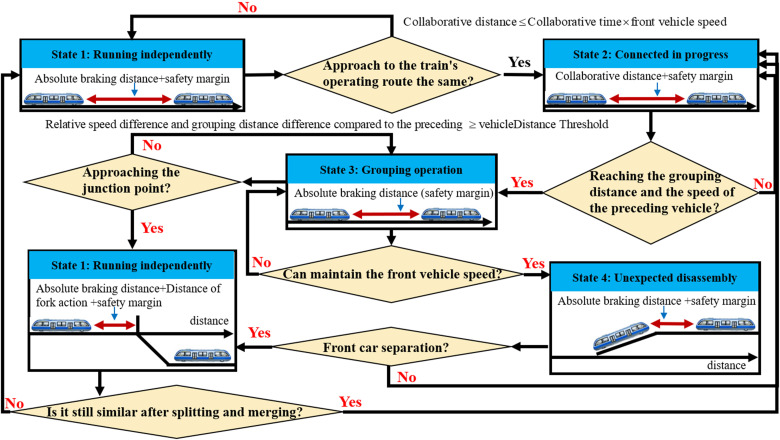
The VC process state flow of MRT vehicles.

The state transition process of VC is illustrated in Fig 4. The decoupling process of MRT vehicles within a virtual formation involves several critical phases. The overall is predominantly influenced by factors such as the performance of the Automatic Train Operation (ATO) system, vehicle dynamics, turnout operation efficiency, and the level of coordination between leading and following vehicles. For instance, during the transition from State 1 to State 2, it is essential to ensure that the following vehicle can synchronize its speed with the leading vehicle within the specified coordination time under varying track conditions. This not only requires low-latency, low-packet-loss communication and high-reliability between the two automated transit vehicles, but also demands that the ATO system possess fast processing capabilities and that the vehicle can promptly respond to control commands. Even under complex track conditions such as uphill and downhill, the following vehicle must still complete synchronization of speed and acceleration/deceleration rates with the leading vehicle within the designated time frame. During the transition from State 3 to State 5, the response speed and reliability of switch operations become critical factors influencing the efficiency of vehicle formation. Given the inherent failure probability associated with conventional track switches, the stability of this process is particularly important. In practice, the transition often involves passing through an unanticipated disassembly state, wherein the following vehicle decelerates, or the leading vehicle accelerates to proactively increase the inter-vehicle distance. This adjustment facilitates the merging or diverging operation at the switch, thereby ensuring continuity and safety in system operation.

Based on the above state transition process, a detailed analysis of typical application scenarios of VC technology in an intelligent connected monorail transit system can be conducted. Depending on different operational requirements, VC can be extended to various representative scenarios, including in-station coupling, tracking-based coupling, out-of-station decoupling, and in-operation decoupling. For each scenario, the control logic, key technical requirements, and operational constraints can be systematically examined in the context of actual working conditions.

#### 3.1.1. In-station vehicle coupling.

**Fig 5:**
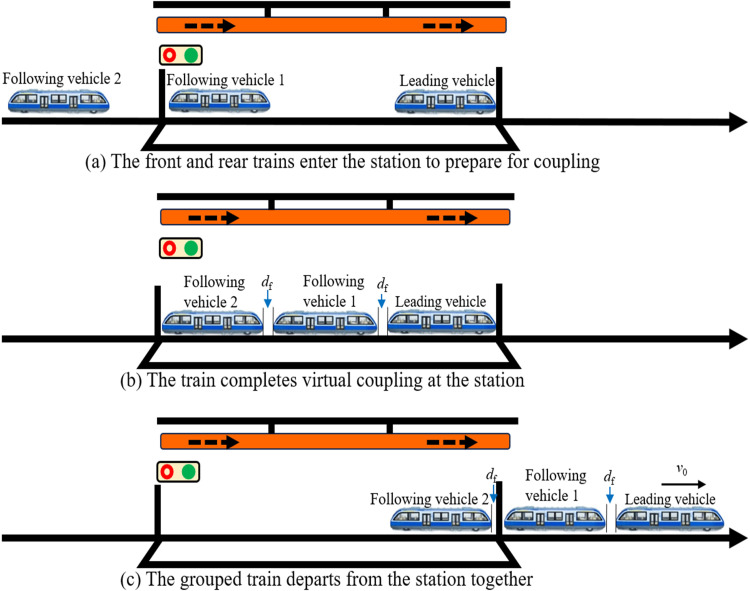
MRT vehicles entry coupling scene.

During peak passenger flow periods, or when the system receives real-time passenger surge predictions at a specific station based on big data analytics, in-station vehicle coupling is typically employed to meet increased capacity demands. As illustrated in Fig 5(a), the typical operation process involves the lead vehicle arriving and stopping at the station, while the following vehicles assigned for coupling exit from a reversing track or depot and enter the platform area for coupling. Once all the following vehicles have arrived, virtual formation is executed at the station according to signal instructions, with an inter-vehicle spacing of $d_{f}$, as shown in Fig 5(b). After completing the formation in accordance with the timetable, the transit vehicles depart in a virtually coupled state, maintaining a uniform speed *v*_0_, as depicted in Fig 5(c). In the in-station coupling scenario, to ensure the smooth execution of the coupling process, it is necessary to account for the inter-vehicle spacing $d_{f}$ between transit vehicles. This involves appropriately extending the dwell time of the lead vehicle at the station while simultaneously increasing the running speed of the following vehicles in the preceding section to enable them to catch up with the lead vehicle.

#### 3.1.2. Tracking-based vehicle coupling.

During off-peak periods, rail vehicles typically operate as independent formations along the line. If a sudden surge in passenger demand occurs at a particular station, the system must promptly initiate a coupling operation before the vehicles arrive at the station to rapidly enhance transport capacity. In this case, individual transit vehicles traveling on the line can perform VC formations in accordance with signaling instructions. Assuming two transit vehicles are operating between two stations with an initial spacing of Δd, where the lead vehicle travels at speed $v_{L}$ and the following vehicle at speed $v_{F}$. A VC operation is triggered once both vehicles simultaneously receive the coupling command, the vehicle ahead is designated as the lead vehicle, and the one behind as the following vehicle. During the coupling process, key factors such as vehicle positions, operating speeds, and relative velocity should be carefully considered. Based on the speed difference between the two vehicles, tracking-based coupling can be divided into two scenarios: accelerated coupling, where the lead vehicle travels faster than the following vehicle, and decelerated coupling, where the following vehicle travels faster than the lead vehicle. The following sections provide a detailed analysis of each scenario.

(1) Accelerated coupling scenario

**Fig 6:**
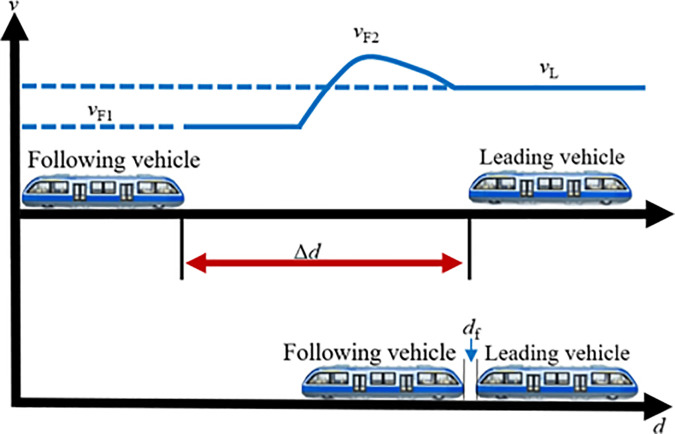
MRT vehicles acceleration coupling scene.

When $v_{L}>v_{F}$, the following vehicle must accelerate to catch up with the lead vehicle, constituting the accelerated coupling scenario. When operating independently on the track, the inter-vehicle distance Δd is typically greater than the required coupling distance $d_{f}$, as sufficient safety margins are needed to accommodate speed variations. As illustrated in Fig 6, once the two vehicles traveling on the same line reach a predetermined decision point and the spacing satisfies the coupling conditions, the following vehicle first accelerates from $v_{F1}$ to a higher speed $v_{F2}$ to reduce the inter-vehicle gap. It then immediately decelerates so that Δd becomes exactly equal to $d_{f}$, while its speed $v_{F2}$ decreases to match the lead vehicle’s speed $v_{L}$. At this moment, the two transit vehicles complete the VC and proceed toward the station at the common velocity $v_{L}$.

(2) Decelerated coupling scenario

**Fig 7:**
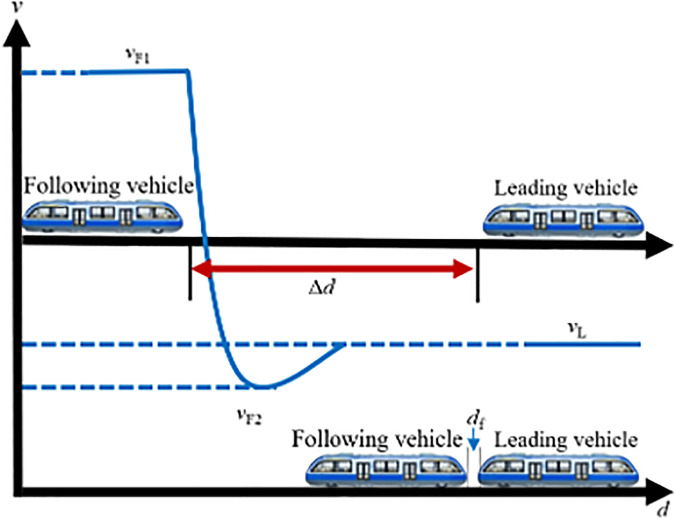
MRT vehicles deceleration coupling scene.

When $v_{L}<v_{F}$, the following vehicle reduces its speed to adjust the relative braking distance, thereby enabling VC with the lead vehicle, this is referred to as the decelerated coupling scenario, as illustrated in Fig 7. In this case, the following vehicle first decelerates from $v_{F1}$ to a lower speed $v_{F2}$, allowing the inter-vehicle distance to decrease gradually. Once the spacing is sufficiently reduced, the following vehicle immediately accelerates again. When the inter-vehicle distance Δd equals the target coupling distance $d_{f}$, the following vehicle increases its speed from $v_{F2}$ to $v_{L}$ for matching the leading vehicle’s speed, completing the VC process. From this point onward, the two transit vehicles travel in a synchronized manner toward the station at the same speed $v_{L}$.

#### 3.1.3. Out-of-station vehicle decoupling.

**Fig 8:**
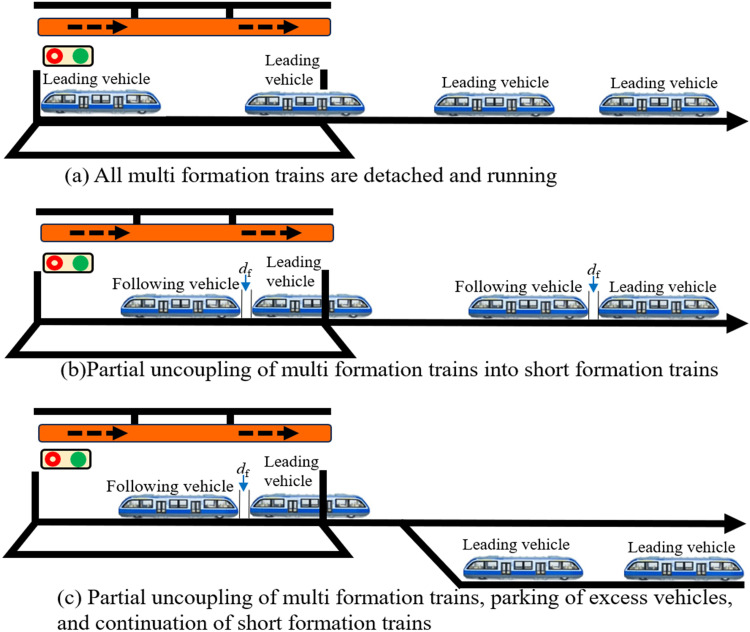
MRT vehicles exit the decoupling scene.

When passenger demand decreases or is expected to decline, multi-unit trainsets can be decoupled at the platform. The decoupling process is straightforward, requiring only a decoupling command to the vehicles. Typically, upon arrival at a station, the entire long formation can be split into independent vehicles, each departing sequentially as a lead vehicle, as shown in Fig 8(a). When passenger volume declines from peak to off-peak but remains above low-peak levels, only partial decoupling is required to form multiple short formations for continued operation, as illustrated in Fig 8(b). During extremely low passenger volumes or near-service-termination periods, only essential vehicles remain in operation. Remaining vehicles are decoupled and parked in turnout sections. Operating vehicles may depart after preceding vehicles enter the turnout, as shown in Fig 8(c). Stored vehicles requiring turnaround or entry into maintenance centers may be rerouted for departure once the turnout section becomes available.

#### 3.1.4. In-operation decoupling.

When passenger demand decreases, the rail transit system may decouple a multi-vehicle formation operating under VC to avoid excess capacity and resource waste. For safety considerations, the preferred moment for decoupling is after the formation has stopped at the nearest station. However, unexpected conditions may require decoupling while the vehicles are still in motion. For a two-vehicle virtually coupled formation traveling with a nominal inter-vehicle distance, such conditions typically fall into two categories. Below are separate analyses:

(1) Passive decoupling due to excessive spacing

**Fig 9:**
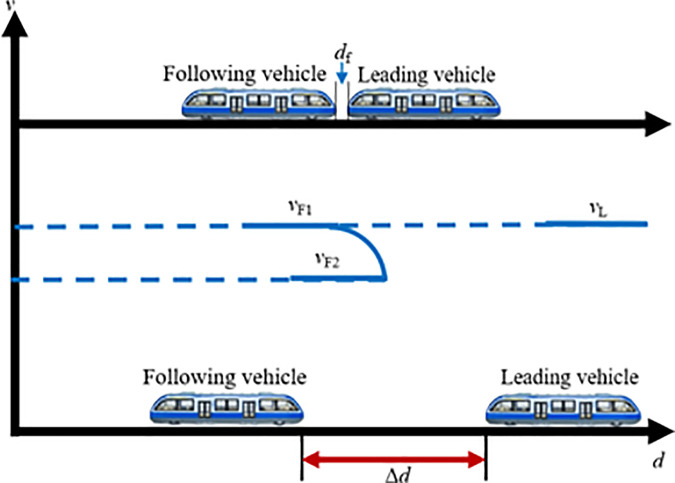
Passive decoupling scene with excessively long spacing.

When $\Delta d>d_{f}$, the system detects an abnormal inter-vehicle distance, it automatically determines that the VC is no longer valid and triggers a decoupling command. This scenario is referred to as passive decoupling due to excessive spacing. At the moment the decoupling signal is issued, the speed of the following vehicle $v_{F}$ is necessarily less than that of the lead vehicle $v_{L}$. As illustrated in Fig 9, a two-vehicle formation operating under VC encounters an unexpected situation in which the following vehicle slows down from $v_{F1}$ to a lower speed $v_{F2}$. This causes the inter-vehicle distance to increase gradually until it exceeds the maximum allowable coupling threshold, resulting in an automatic system-triggered decoupling. Once decoupling is completed, the leading vehicle continues to operate independently at speed $v_{L}$, while the following vehicle proceeds at speed $v_{F2}$. The two vehicles exit the VC state and resume operation as independent units.

(2) Active decoupling due to insufficient spacing

**Fig 10:**
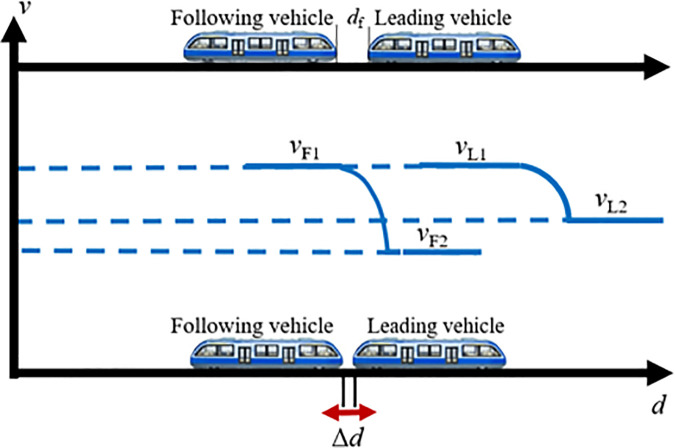
Active disconnection scene with insufficient spacing.

When $\Delta d<d_{f}$ (assuming the minimum collision-avoidance distance is equal to the nominal coupling distance $d_{f}$, though in practice it is typically smaller), the system immediately identifies a collision risk and triggers the active decoupling mechanism. This scenario is referred to as active decoupling due to insufficient spacing. At the moment active decoupling is initiated, the speed of the following vehicle $v_{F}$ is necessarily greater than that of the lead vehicle $v_{L}$. As illustrated in Fig 10, a virtually coupled two-vehicle formation encounters a situation where the lead vehicle unexpectedly decelerates from $v_{L1}$ to a lower speed $v_{L2}$. As a result, the inter-vehicle distance decreases until it falls below the minimum safe separation defined by the system. To prevent a rear-end collision, the following vehicle proactively decouples from the leading vehicle. After decoupling, it initiates an emergency braking maneuver, reducing its speed to a value lower than the current speed of the lead vehicle.

### 3.2. Collision scenario analysis

**Fig 11:**
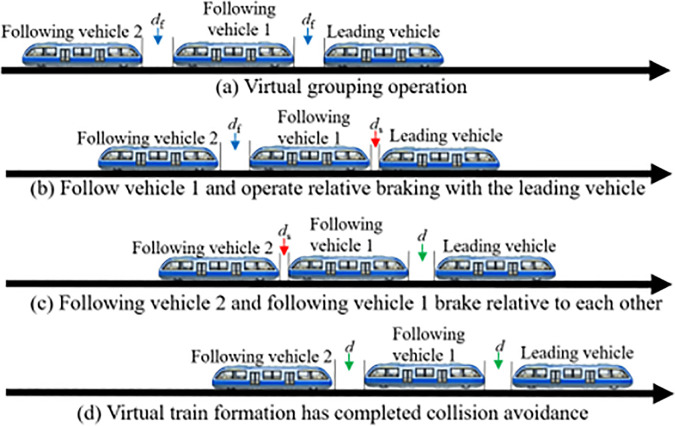
Collision avoidance diagram for MRT vehicles in VC mode.

To meet the safety requirements of VC operation, a collision avoidance control strategy is developed based on the Relative Distance Braking Mode (RDBM), using the minimum safe distance between transit vehicles as the triggering condition. When a monorail vehicle experiences a reduction in inter-vehicle distance due to speed variation- such that the spacing falls below the predefined safety threshold- the system immediately activates the emergency braking mechanism of the following vehicle to prevent a rear-end collision. Fig 11 illustrates the conceptual diagram of the collision avoidance strategy under VC mode.

Before developing collision avoidance strategies for rail transit vehicles, it is essential to first establish representative collision scenarios and analyze the underlying causes of potential collisions in virtually coupled formations. During operation, the most probable causes of the control system are significant delays in vehicle-to-ground or V2V communication. Such failures can lead to abnormal fluctuations in vehicle speed, ultimately disrupting the stability of the VC formation. From an operational perspective, when virtually coupled vehicles receive commands to enter or depart from a station, the rapid variation in formation speed can easily introduce safety risks. In addition, if a transit vehicle encounters an obstacle on the track during operation, it must initiate emergency braking to come to a stop. Based on these typical situations, this study defines and constructs three critical collision scenarios: acceleration during station departure, deceleration during station arrival, and obstacle-induced collision during operation.

#### 3.2.1. Acceleration during station departure.

**Fig 12:**
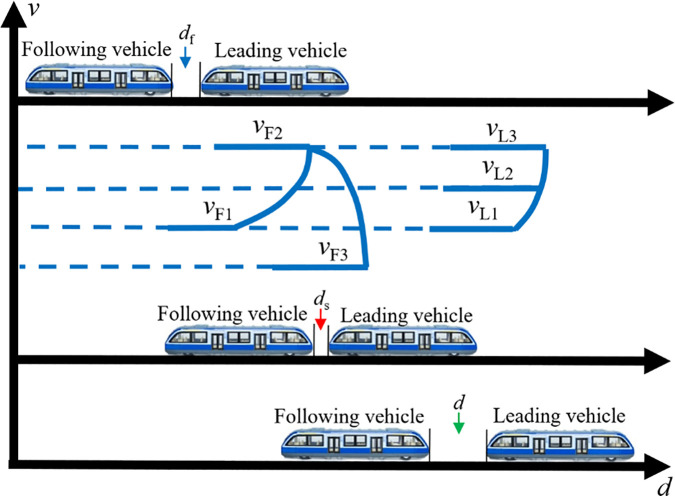
The scene of a collision involving MRT vehicles accelerating out of the station.

After completing the VC at the station, the train begins accelerating to depart. Suppose the coupled train accelerates to a target speed $v_{\text{1}}$, with the leading vehicle moving at $v_{L1}$, the following vehicle at $v_{F1}$, and $v_{L1}=v_{F1}$. Due to system faults such as excessive latency in vehicle-to-ground or inter-vehicle communication, inconsistent acceleration may occur within the virtual formation, leading to a speed mismatch. At time *t*, if the leading vehicle’s speed exceeds that of the following vehicle, the inter-vehicle distance gradually increases, triggering a passive decoupling due to excessive spacing. Once communication stabilizes, the system may switch to an acceleration coupling mode to re-establish the virtual link. Conversely, if the lead vehicle’s speed is lower than the following vehicle’s at time *t*. Due to $v_{L2}<v_{F2}$, the gap between them decreases for posing a collision risk is posed. When the following vehicle detects that the inter-vehicle distance has decreased to the minimum safety threshold $d_{s}$, an emergency braking is immediately triggered. The following vehicle decelerates while the lead vehicle continues to accelerate toward its target speed $v_{L3}$. Afterward, the gap shrinks, but the distance begins to widen as braking continues. Once the spacing exceeds the safety threshold again, braking ceases, and the collision is successfully avoided. This departure acceleration collision avoidance scenario is illustrated in Fig 12. If the vehicle performance is not affected during subsequent inspections and there is still a need for VC, the corresponding acceleration coupling scenario can be switched to, and VC can be performed again.

#### 3.2.2. Deceleration during station arrival.

**Fig 13:**
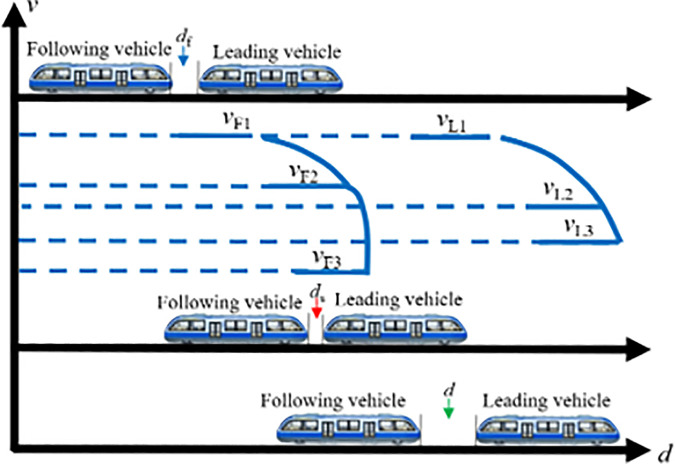
The scene of a collision involving MRT vehicles accelerating out of the station.

When MRT vehicles operate normally and approach a station, a gradual deceleration process naturally takes place. Suppose that at this moment, the virtual formation of MRT vehicles is travelling at normal speed $v_{\text{1}}$; the speeds of the leading and following vehicles are $v_{L1}$ and $v_{F1}$, respectively, with $v_{L1}=v_{F1}$. The formation of the MRT vehicles receives a signaling command to begin deceleration and prepare for station entry. Similar to the acceleration departure scenario, due to system faults such as excessive latency in vehicle-to-ground or inter-vehicle communication, which causes inconsistent deceleration among different vehicles in the virtual trainset, ultimately introducing speed differentials. As in the acceleration case, the speed of the leading car is lower than that of the following car, and as the inter-vehicle distance decreases, the risk of collision increases significantly. Once the front-end of the following car detects that the gap to the rear-end of the leading car has narrowed to the predefined safety distance $d_{s}$, it triggers an emergency braking procedure on the basis of normal deceleration braking. The deceleration rate of the following car thereby increases significantly, while the leading car continues to decelerate as planned according to the control signal until reaching $v_{L3}$. The emergency braking ensures that the speed of the following car rapidly drops to $v_{F3}$ within a very short period. The inter-vehicle distance d continues to shrink slightly before increasing again until it exceeds the safety threshold $d_{s}$, at which point braking is discontinued, and a collision is successfully avoided. This entire process is illustrated in Fig 13. If subsequent diagnostics confirm that the vehicle’s performance remains unaffected and VC is still required, the system can be switched to the station coupling scenario, and VC can be performed again.

#### 3.2.3. Obstacle-induced collision during operation.

**Fig 14:**
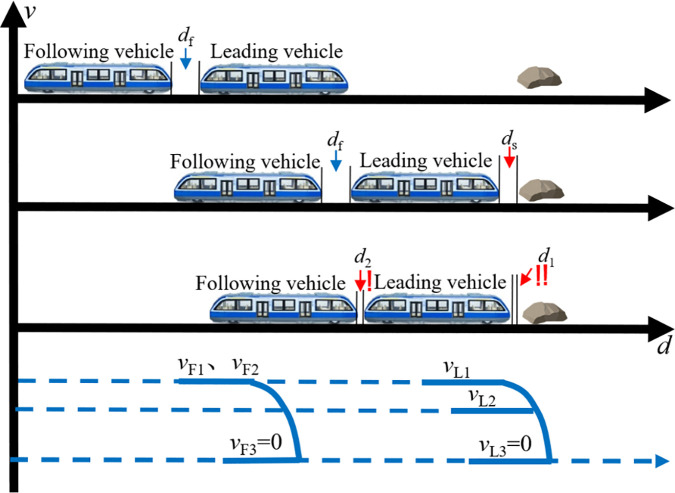
The scene where MRT vehicles collide with an obstacle during operation.

Assuming that the virtual trainset is operating at normal speed $v_{\text{1}}$ along the track, where the leading vehicle travels at speed $v_{L1}$ and the following vehicle at speed $v_{F1}$ as shown in Fig 14, an unexpected obstacle suddenly appears ahead. In this scenario, the leading vehicle is unable to detect the obstacle via visual or signal-based recognition systems and can only perceive its distance from the obstacle through onboard proximity sensors. As the virtual trainset continues to move forward, the distance between the leading vehicle and the obstacle decreases. When this distance reduces to the predefined safety threshold $d_{s}$, the leading vehicle immediately initiates emergency braking, causing its speed to drop rapidly to $v_{L2}$. However, the following vehicle, which remains within the VC range $d_{s}$ and has not yet reached its own safety distance from the leading vehicle, does not initiate braking and continues to travel at a constant speed ($v_{F2}=v_{F1}$). In the subsequent moment, while the leading vehicle continues braking, the following vehicle maintains its speed. Once the distance between the front of the following vehicle and the rear of the leading vehicle decreases to the safety distance $d_{s}$, the following vehicle also engages emergency braking and decelerates rapidly. This braking process continues until all vehicles in the trainset have come to a complete stop, awaiting assistance from the track maintenance department.

### 3.3. Virtual coupling control strategy

The operation of virtual-coupled trainsets in urban rail transit primarily involves three fundamental operating modes: traction, coasting, and braking. The corresponding control objectives are as follows: (1) the leading vehicle is capable of accurately tracking control centers, cloud platforms, or preset speed-distance curves in a short period of time; (2) the following vehicle should precisely and rapidly track the speed of the leading vehicle, maintaining a consistent velocity and a desired inter-vehicle distance $d_{f}$. The control strategies for these three operating modes are described as follows:

(1) Traction condition

During operation, scenarios such as the start-up phase and the acceleration phase are categorized as a traction condition. Upon receiving the corresponding speed command, the MRT vehicle generates the required tractive force to accelerate accordingly. While operating under traction mode, the MRT vehicle is simultaneously subjected to tractive force and resistive force, acting in opposite directions. When in traction, the vehicle accelerates from an initial speed of 0 to a target velocity $v_{max}$. Assuming the total acceleration time is T, and the acceleration distance is S, the motion during the traction phase can be described as follows:


$$\left\{ \begin{array}{c} @c@s_{a}=\frac{v_{max}^{\text{2}}-v_{\text{0}}^{\text{2}}}{2a}\\ t_{a}=\frac{v_{max}-v_{\text{0}}}{a} \end{array}\right.$$ (1)

In the above expressions, $s_{a}$ refers to the distance traveled by the vehicle during the traction phase, $t_{a}$ is the corresponding duration, and a denotes the acceleration experienced during this phase.

(2) Coasting condition

The coasting condition refers to the operating phase in which the MRT vehicle is subject only to resistive forces, without the influence of tractive or braking forces. In this stage, the vehicle can be considered to travel at a nearly constant velocity. Typically, during the operation of urban rail transit vehicles, the coasting condition serves as a transitional state, occurring between the traction and braking phases. The dynamics during the coasting phase can be described as follows:


$$t_{c}=\frac{s_{c}-s_{a}}{v_{max}}$$ (2)

Where $s_{c}$ indicates the position of the urban rail vehicle at the conclusion of the coasting phase, while $t_{c}$ denotes the duration of coasting.

(3) Braking condition

The braking condition refers to the phase in which the urban rail vehicle actively applies braking force to decelerate. This condition is typically employed to ensure precise stopping, enable emergency braking, and fulfill various safety protection requirements. The dynamics during the braking phase can be described as follows:


$$\left\{ \begin{array}{c} @c@s_{b}=\frac{v_{max}^{\text{2}}-v_{\text{0}}^{\text{2}}}{2a}\\ t_{b}=\frac{v_{max}-v_{\text{0}}}{a} \end{array}\right.$$ (3)

Where $s_{b}$ denotes the travel distance of the urban rail vehicle during the braking phase, $t_{b}$ represents the duration of this phase, and a is the braking deceleration. Therefore, the braking phase can be described as:


$$\left\{ \begin{array}{c} @c@S=s_{c}+s_{b}\\ T=t_{a}+t_{b}+t_{c} \end{array}\right.$$ (4)

In actual operation, urban rail vehicles do not immediately execute the commanded actions at the moment the control system issues a state transition instruction. Due to communication latency, a certain delay often occurs before the operating condition transitions to the target state specified by the command. This period is referred to as the transmission delay. Moreover, after receiving the instruction transmitted through the communication system, the vehicle exhibits a further delay in response due to its inertial characteristics, which is termed the response delay. In addition, energy losses occur during the transmission of power from the vehicle’s motor, resulting in a discrepancy between the commanded tractive or braking force and the actual force delivered. As a result, during both traction and braking conditions, it is necessary to account not only for transmission and response delays, but also for the efficiency of the motor’s actual output.

The traction condition is typically divided into three stages during operation: start-up, retraction, and traction cut-off. The start-up stage refers to the period during which the urban rail vehicle accelerates from a stationary state to the point where the initial traction acceleration begins to decrease, i.e., the traction acceleration increases from zero to a stable value. The retraction stage corresponds to the phase where the vehicle is subjected to traction again, while the traction cut-off stage describes the process during which the vehicle’s speed gradually decreases to zero. Assuming that the transmission delay and response delay correspond to $T_{\text{1}}$ and $T_{\text{2}}$, respectively, the following applies in the start-up stage: When the time is less than $T_{\text{1}}$, the step command signal for start-up has not yet been transmitted to the vehicle, and the acceleration remains zero. Conversely, the vehicle successfully receives the start-up command and outputs the traction force corresponding to the desired acceleration to accelerate. However, due to mechanical transmission losses, the actual acceleration applied to the vehicle deviates from the target acceleration, and thus, an output efficiency factor $P_{\text{1}}$ should be introduced. Based on the above, the acceleration output formula during the traction start-up phase can be expressed as:


$$a_{in}=\left\{ \begin{array}{c} @c@0,t\leq T\\ a\left[1-exp\left(-\frac{t-T_{\text{1}}}{T_{\text{2}}}\right)\right],t>T_{\text{1}} \end{array}\right.$$ (5)

Based on the aforementioned basic operating conditions of the urban rail vehicles, any vehicle within the virtual trainset can obtain the complete state information of all members in the formation. However, considering the vehicle length, effective and reliable V2V communication is only feasible between adjacent vehicles. Therefore, this paper models the virtual trainset system as a graph M=(V,E), where V={1,2,3,4,5,6,⋯n} denotes the set of all vertices representing the MRT vehicles, and $E=\left\{e_{\text{1}},e_{\text{2}},e_{\text{3}},\cdots ,e_{m}\right\}$ denotes the set of edges representing V2V communication links. In the set of edges, the edges composed of vertices i and j are represented as $e_{ij}=\left(v_{i},v_{j}\right)$, where i is the starting vertex and j is the ending vertex by default. The adjacency matrix of the graph M is denoted as $A_{ij}=[a_{ij}{]}_{n\times n}$, specifically:


$$a_{ij}=\left\{ \begin{array}{c} @c@w_{ij},\left(v_{i},v_{j}\right)\in E\\ 0,other \end{array}\right.$$ (6)

Generally, the values of $w_{ij}$ are either 0 or 1. Considering the dynamic variability of the V2V communication link quality, $w_{ij}$ can be extended to represent the link quality. When $a_{ij}=1$, it indicates that there exists a unique edge between vertices i and j, and the link quality is sufficient to support the virtual trainset communication requirements without additional demand. Otherwise, the communication link between the vehicles is abnormal, leading to impaired information transmission, such as increased latency, packet loss, and other phenomena that degrade control performance.

The Laplacian matrix characterizes the algebraic connectivity of the graph [35], while also describing the mapping relationship between urban rail vehicles and communication links at the same time. $L\left(M\right)=\left|l_{ij}\right|$ is defined as follows:


$$l_{ij}=\left\{ \begin{array}{c} @c@-a_{ij},i\neq j,j\in N_{i}\\ \sum_{j\in N_{i}}a_{ij},i=j\\ 0,other \end{array}\right.$$ (7)

In fact, the Laplacian matrix can be expressed as $L=BB^{T}$. Assuming that the V2V communication links are normal, the adjacency matrix $A_{ij}=[a_{ij}{]}_{n\times n}$ is unweighted, with elements being either 0 or 1. Let there be a matrix $B\in R^{n\times m}$, where m denotes the number of edges in the graph M=(V,E) and n is the number of vertices. Then the matrix $B\in R^{n\times m}$ is represented as:


$$[B{]}_{k}=\left\{ \begin{array}{c} @c@+1,i\text{ is starting point of edge }e_{k}\\ 0,other\\ -1,i\text{ is ending point of edge }e_{k} \end{array}\right.$$ (8)

The operational environment of urban rail transit is complex and dynamic. The inter-vehicle spacing constantly changes, and the performance of V2V communication links is influenced by relative speed and distance. Consequently, the link quality exhibits temporal and spatial variations, with metrics such as latency, packet loss, and throughput fluctuating accordingly. This paper assumes that under a coexistence model of V2V and V2I communication, communication metrics like packet loss and latency meet the minimum requirements for virtual trainset operation. Considering that the relative speed and relative distance between urban rail vehicles dynamically change, we employ the classical artificial potential field method to theoretically analyze and validate the virtual trainset performance under different operating conditions. The safety of VC relies on controlling the inter-vehicle spacing to prevent rear-end collisions, while efficiency improvement also depends on maintaining appropriate spacing to avoid excessively large gaps. The artificial potential field method constructs potential functions based on the relative kinematic parameters between vehicles, ensuring that when the spacing is too large, vehicles are attracted closer, and when the spacing is too small, they are repelled apart [32]. Therefore, the VC control model for urban rail vehicles is formulated as follows:


$$\begin{array}{c} u_{ix}\left(x_{i},x_{j}\right)=-k_{\text{1}}\sum_{j=1}^{n}a_{ij}\nabla In\left[cos\left(x_{i}-x_{j}-d_{f}\right)\right]\\ \text{=}-k_{\text{1}}\sum_{j=1}^{n}a_{ij}tanh(x_{i}-x_{j}-d_{f}) \end{array}$$ (9)

$$\begin{array}{c} u_{iv}\left(v_{i},v_{j}\right)=-k_{\text{2}}\sum_{j=1}^{n}a_{ij}\nabla In\left[cos\left(v_{i}-v_{j}\right)\right]\\ \text{=}-k_{\text{2}}\sum_{j=1}^{n}a_{ij}tanh(v_{i}-v_{j}) \end{array}$$ (10)

$$u_{iAPF}\left(x_{i},x_{j},v_{i},v_{j}\right)=u_{ix}\left(x_{i},x_{j}\right)+u_{iv}(v_{i}-v_{j})$$ (11)

In the equation, $k_{\text{1}}$ is defined as the gain of the position potential field, by which the vehicle’s capability to regulate its position and maintain the desired spacing $d_{f}$ is determined, and $k_{\text{2}}$ is defined as the gain of the velocity potential field, through which the vehicle’s ability to attenuate speed differences and achieve velocity coordination is governed; $x_{i}$ and $x_{j}$ denote the current positions of urban rail vehicles i and j, respectively; $v_{i}$ and $v_{j}$ represent the current velocities of vehicles i and j, respectively; and $d_{f}$ denotes the VC distance within the trainset.

### 3.4. Algorithm design

**Fig 15:**
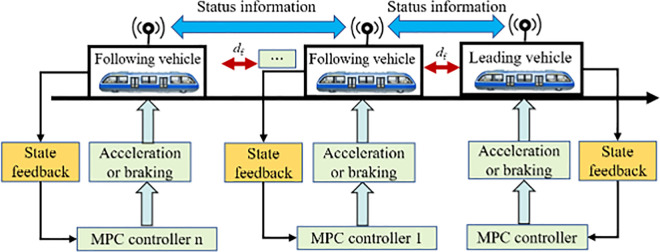
Architecture of a virtual connected distributed model predictive control algorithm.

Based on the aforementioned model, a distributed model predictive control (DMPC) algorithm is employed to address the control problem of VC. The architecture of the controller is illustrated in Fig 15. Leveraging wireless communication technologies, adjacent urban rail vehicles are able to share real-time information such as speed, position, and acceleration. Using both its own state parameters and those of the preceding vehicle, each controller solves a local optimization problem to obtain the optimal control law for the vehicle. At each sampling instant, the controller continuously updates vehicle states and recalculates the control input until the entire VC convoy reaches a stable operating state.

To facilitate the design of distributed controllers, it is first assumed that the position $x_{\text{1}}$ of the leading vehicle has a first derivative $v_{\text{1}}$, representing its velocity, and a bounded second derivative $a_{\text{1}}$, satisfying $sup\left|v_{\text{1}}\right|\leq \theta_{\text{1}}$， $sup\left|a_{\text{1}}\right|\leq \theta_{\text{2}}$, where $\theta_{\text{1}}$、 $\theta_{\text{2}}$ are all bounded and well-defined. Based on the communication topology of the virtually coupled urban rail vehicles shown in the figure above, the specific form of the control algorithm is expressed as follows:


$$v_{i}=-\beta_{\text{1}}^{\frac{\text{1}}{b_{\text{2}}}}sig^{\frac{\text{1}}{b_{\text{2}}}}\left[\phi_{i}+\alpha_{\text{1}}sig^{\frac{\text{1}}{b_{\text{1}}}}\left(K_{i}\right)\right]-k_{\text{1}}sign\left(\phi_{i}\right)$$ (12)

$$a_{i}=-\beta_{\text{1}}^{\frac{\text{1}}{b_{\text{2}}}}sig^{\frac{\text{1}}{b_{\text{2}}}}\left[K_{i}+\alpha_{\text{1}}sig^{\frac{\text{1}}{b_{\text{1}}}}\left(K_{i}\right)\right]-k_{\text{2}}sign\left(K_{i}\right)$$ (13)

Let $v_{i}$、 $a_{i}$ denote the estimated velocity and acceleration of the i−th vehicle, respectively. The parameters $k_{\text{1}}$ and $k_{\text{2}}$ are positive constants, while $\alpha_{\text{1}}>0$、 $\beta_{\text{1}}>0$， $1<b_{\text{2}}<2$， $b_{\text{2}}<b_{\text{1}}$. The $\phi_{i}$、 $K_{i}$ are determined by the following equations:


$$\phi_{i}=\sum_{j=i-1}^{i+1}b_{ij}(x_{i}-x_{j})+c_{i}(x_{i}-x_{\text{1}}),\;i,j\in n$$ (14)

$$K_{i}=\sum_{j=i-1}^{i+1}a_{ij}(v_{i}-v_{j})+c_{i}(v_{i}-v_{\text{1}}),\;i,j\in n$$ (15)

Where $x_{i}$ and $x_{j}$ denote the estimated positions of the i−th and j−th vehicles, respectively. While $v_{i}$ and $v_{j}$ represent the corresponding estimated velocities.

For the control algorithm applied to vehicle formations operating under VC, the convergence time T required for a successful coupling of the train formation satisfies:


$$T\leq T_{\text{0}}=\frac{2b_{\text{2}}\beta_{\text{1}}^{\frac{\text{1}}{b_{\text{2}}}}}{\varphi_{\text{1}}^{\frac{\text{1}}{b_{\text{2}}}}(b_{\text{2}}-1)}+\frac{2b_{\text{2}}\beta_{\text{1}}^{\frac{\text{1}}{b_{\text{2}}}}}{\left(\alpha_{\text{1}}\varphi_{\text{2}}{)}^{\frac{\text{1}}{b_{\text{2}}}}(b_{\text{1}}-b_{\text{2}}\right)}$$ (16)

Where $\varphi_{\text{1}}=N^{\frac{1-b_{\text{2}}}{\text{2}}}(2\lambda_{min}(Q{)}^{\frac{b_{\text{2}}+1}{\text{2}}})$， $\varphi_{\text{2}}=N^{\frac{2-b_{\text{1}}-b_{\text{2}}}{\text{2}}}(2\lambda_{min}(Q{)}^{\frac{b_{\text{1}}+b_{\text{2}}}{\text{2}}})$， $\lambda_{min}$ denotes the minimum eigenvalue of the matrix Q.

**Fig 16:**
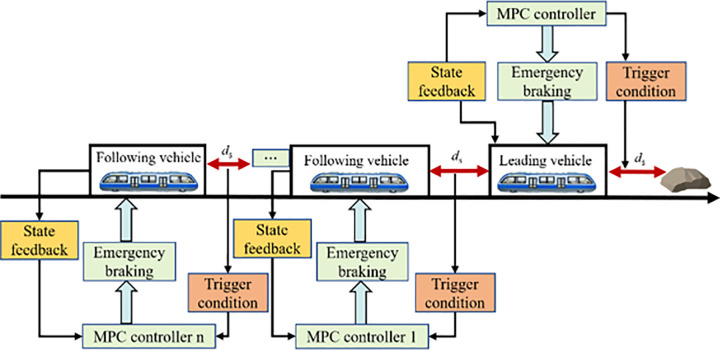
Architecture of the collision avoidance active model predictive control algorithm.

To address the emergency braking and collision avoidance control problem in virtually coupled MRT vehicle formations, a centralized MPC scheme is proposed. The control architecture of the system is illustrated in Fig 16. Within the virtual formation, all MRT vehicles transmit their state information, such as velocity, position, and acceleration, to a centralized MPC controller via internal communication networks. Based on the inter-vehicle distance as a triggering condition, the controller autonomously determines whether emergency braking should be initiated, thereby ensuring the safe operation of the train formation. The following section details the design process of the centralized MPC controller.

Assuming a discrete sampling time of Δt，the discretized state-space model is written as:


$$d_{s}=d_{b}+d_{m}$$ (17)

Where k denotes the discrete sampling instant; $s_{i}\left(k\right)$ is the current position of the i−th urban rail vehicle at sampling instant k (m); $v_{i}\left(k\right)$ is the current velocity of the i−th vehicle at sampling instant k (m/s); $a_{i}\left(k\right)$ is the current acceleration of the i−th vehicle at sampling instant k (m/s²); $u_{i}\left(k\right)$ represents the acceleration command issued by the braking system for the i−th vehicle at sampling instant k (m/s²); $w_{i}\left(k\right)$ is the acceleration due to basic resistance of the i−th vehicle at sampling instant k (m/s^2^); $g_{i}\left(k\right)$ is the acceleration due to additional resistance at sampling instant k (m/s²).

When the virtually coupled trainset operates along the railway line, its dynamic behavior must conform to constraints imposed by the inherent characteristics of each vehicle and the conditions of the railway infrastructure. The following constraints are incorporated into the model:


$${u_{i}}^{min}\leq u_{i}(k+j|k)\leq {u_{i}}^{max}$$ (18)

$$0\leq v_{i}(k+j|k)\leq v_{lim}\left(x_{i}\right)$$ (19)

$$s_{i-1}(k+j|k)-s_{i}(k+j|k)\geq s_{m}-\frac{v_{i-1}(k{)}^{\text{2}}}{2u_{i-1}^{min}}+\frac{v_{i}(k{)}^{\text{2}}}{2u_{i}^{min}}\text{\textbackslash{}hspace\{0.5em}\;\;\;\;\;\;\;\;\;\;\;\;\;\;\;\;\;\;\;\;\;\;\;\;\;\;\;\;\;\;\;\;\;\;\;\;\;\;\;\;\;\;\;\;\;\;\;\;\;\;\;\}$$ (20)

Where ${u_{i}}^{min}$ denotes the maximum deceleration of the i−th vehicle during emergency braking (m/s^2^); ${u_{i}}^{max}$ denotes the maximum traction acceleration during vehicle acceleration (m/s²); $v_{lim}\left(x_{i}\right)$ is the maximum allowable operating speed of the urban rail vehicle at position $x_{i}$ (m/s); and $s_{m}$ is the minimum safety margin required during stopping (m).

Based on the above constraints, the optimization problem for emergency collision avoidance braking in virtually coupled urban rail vehicles can be formulated as follows:


$$u_{des\left(k\right)}minJ\left(k\right)=\sum_{j=1}^{M}\sum_{i=2}^{N}m_{i}[v_{i}-1(k+j|k)-v_{i}(k+j|k){]}^{\text{2}}$$ (21)

This assumption is justified as the resistance variation can be neglected within the relatively short prediction interval, since emergency braking on straight tracks is a transient process. The present study focuses exclusively on collision avoidance under straight track conditions, without considering curved track effects. After the above optimization simplifications, the problem formulated in Equation (21) can be transformed into a quadratic programming (QP) problem, which includes several linear constraints and can be efficiently solved by various classical algorithms. Assuming the optimal control sequence obtained from the optimization problem in Equation (21) is:


$$d_{s}=d_{b}+d_{m}$$ (22)

The first element of the control sequence $u_{i,des}\left(k|k\right)$ is applied to control the emergency braking of the MRT vehicle. At the next sampling instant, the time index is updated to k=k+1, and the optimization process is repeated, thus implementing a receding horizon control strategy. This iterative process continues until all MRT vehicles have safely decelerated and the inter-vehicle spacing has been restored to a safe distance.

## 4. Experimental results and analysis

### 4.1. Virtual coupling simulation

**Fig 17:**
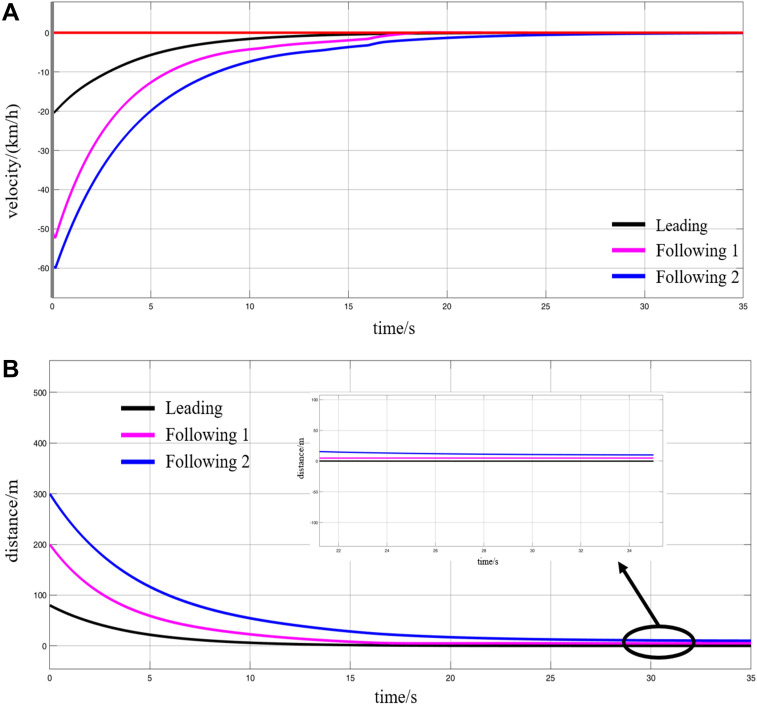
Simulation results of the station entry coupling process, (A) speed variations, (B) displacement variations.

To validate the proposed virtual coupling (VC) theory and control implementation strategy for MRT vehicles, and to demonstrate the superiority of the VC approach, simulations are conducted based on Chongqing Rail Transit Line 3 straddle-type MRT vehicles using MATLAB as the simulation platform. By integrating a multi-body dynamic monorail model, a general driving scenario of VC for MRT vehicles is constructed. The simulation focuses on a virtual trainset composed of three MRT vehicles as well as individual vehicles within the trainset. Since the decoupling process within the VC trainset is relatively straightforward, requiring only communication command signals and not involving complex scenarios, it is not considered in detail in this study. Therefore, this study focuses only on the simulation of VC in two scenarios: single MRT vehicle coupling during station entry and tracking coupling. The process of VC involving multiple trainsets (e.g., multi-trainset to single vehicle, or multi-trainset to multi-trainset coupling) follows the same control principle. From the perspective of cooperative control, each multi-trainset system can be regarded as a single MRT vehicle unit. Based on the representative parameters of driver-cab-equipped vehicles from Chongqing Rail Transit Line 3, a simulation experiment is established to model the station-entry coupling scenario. The mass of each vehicle is set uniformly as M=28600kg, and to approximate real operational conditions, each MRT vehicle is initialized with a specific starting position and velocity. In the station-entry coupling simulation, it is assumed that all three MRT vehicles receive the VC command before entering the station and begin operating under the initial conditions defined in Table 2. The simulation results of the station-entry VC process are shown in Fig 17.

**Table 2:** Initial conditions for the in-station coupling scene.

Application scenario	In-station coupling		
**MRT vehicles**	**Initial speed (km/h)**	**Distance to the station (m)**	**Distance to the preceding vehicle (m)**
**Leading vehicle**	20	80	/
**Following vehicle 1**	52	200	120
**Following vehicle 2**	60	300	100

As shown in the figure, once the MRT vehicles receive the VC command, they begin to decelerate immediately for station approach. The leading vehicle’s speed decreases to 0 at *t* = 15 s, with its position decreasing from the initial 80 m to 0 m, indicating successful docking at the designated station position. The speed of following vehicle 1 reaches 0 at *t* = 17 s, with its position dropping from 200 m to 0 m, indicating that the following vehicle 1 has entered the station and stopped at the corresponding position as instructed, completing the coupling with the leading vehicle. The inter-vehicle spacing $d_{f}$ is controlled at 5 m. The following vehicle 2’s velocity reaches 0 at *t* = 25 s, with its position decreasing from 300m to 0m, indicating that the following vehicle 2 has entered the station and parked at the corresponding position according to the instructions, and has completed the coupling with the following vehicle 1 and the leading vehicle. The grouping $d_{f}$ distance between the following vehicle 2 and the following vehicle 1 is also controlled at 5 m. This marks the successful completion of the entire VC process during the station approach for the three monorail cars.

**Fig 18:**
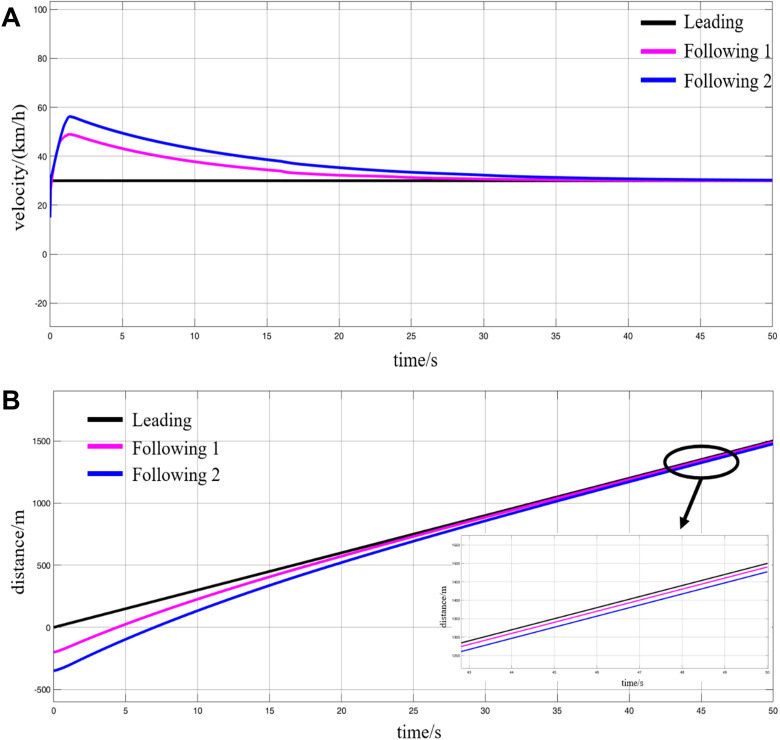
Simulation results of the accelerated coupling process, (A) speed variations, (B) displacement variations.

In the acceleration-based VC simulation for tracking scenarios, it is assumed that a single transit vehicle operating on the line encounters an unexpected situation—such as a sudden surge in passenger demand at the upcoming station. To maximize operational efficiency and ensure timely boarding, the system requires that vehicles form a virtual coupled formation before arriving at the station. In this simulation, three monorail vehicles receive the VC command in advance and begin operating under the initial conditions defined in Table 3. The simulation results are shown in Fig 18.

**Table 3:** Initial conditions for the accelerated coupling scenario.

Application scenario	Accelerated coupling	
**MRT vehicles**	**Initial velocity (km/h)**	**Distance to the preceding vehicle (m)**
**Leading vehicle**	30	0
**Following vehicle 1**	20	200
**Following vehicle 2**	18	100

The simulation results demonstrate that, upon receiving the VC command while operating along the track, the leading vehicle maintains a constant speed, continuing its motion at the original velocity. In contrast, the two following vehicles undergo a brief acceleration phase, during which the inter-vehicle spacing decreases, and their speeds reach the maximum value required for the coupling process. Subsequently, the following vehicles gradually decelerate in a relatively slow manner. Once the following vehicles have successfully closed the gap with the leading vehicle to meet the required VC distance, their speeds converge to match that of the leading vehicle. At this point, the virtual coupled platoon is formed, maintaining an inter-vehicle spacing of 5 m. The three MRT vehicles then proceed together toward the station at a uniform speed of 30 km/h, completing the entire accelerated VC process.

**Fig 19:**
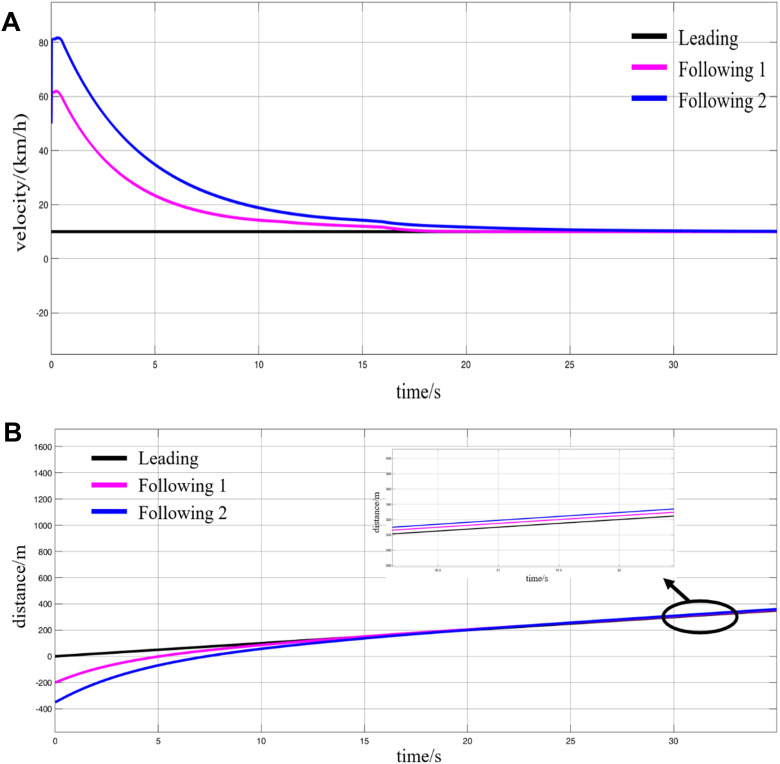
Simulation results of the deceleration coupling process, (A) speed variations, (B) displacement variations.

In the decelerated VC simulation, it is assumed that three MRT vehicles receive the VC command while traveling along the track and begin operation based on the initial conditions specified in Table 4. The corresponding simulation results are presented in Fig 19.

**Table 4:** Initial conditions for deceleration coupling scenarios.

Application scenario	Decelerated coupling	
**MRT vehicles**	**Initial velocity (km/h)**	**Distance to the preceding vehicle (m)**
**Leading vehicle**	10	0
**Following vehicle 1**	60	200
**Following vehicle 2**	80	150

It can be observed that, similar to the accelerated VC scenario, once the MRT vehicles receive the VC command while operating on the track, the leading vehicle maintains a constant velocity, continuing to move forward at its original speed. The following vehicles, on the other hand, are required to travel at speeds higher than that of the leading vehicle while simultaneously applying gradual braking to decelerate. During this process, the inter-vehicle spacing continuously decreases until it reaches the required VC formation distance of 5 m. At this moment, the following vehicles cease braking, and their current speeds reduce precisely to match that of the leading vehicle. Thus, the three MRT vehicles complete the decelerated VC process while in motion and proceed as a coupled formation toward the station at a unified speed of 10 km/h.

### 4.2. Collision avoidance simulation

**Fig 20:**
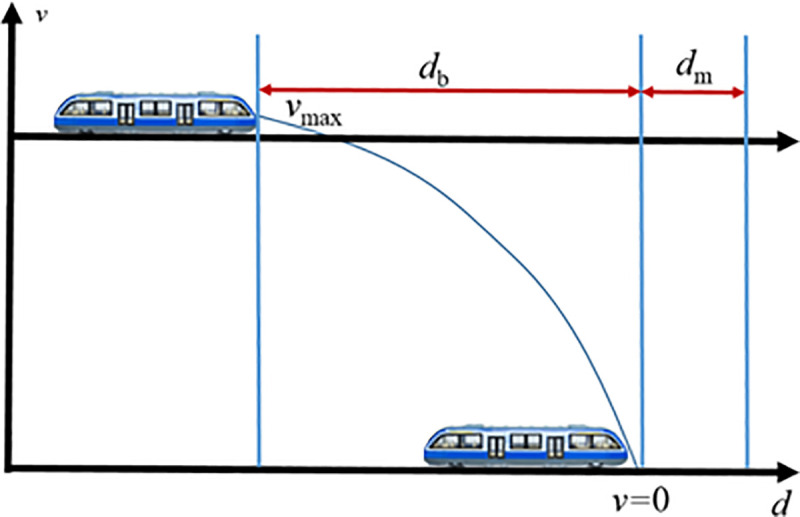
Schematic diagram for setting the safety distance of the virtual formation MRT vehicles' collision avoidance system.

Based on the operational characteristics of intelligent connected monorail vehicles under different VC scenarios, a simulation was conducted to analyze potential collision risks among grouped vehicles in a VC environment. Before the simulation, it is necessary to define a scientifically sound and reasonable safety distance to serve as the warning threshold, which triggers the vehicle’s onboard collision avoidance system. This setup significantly enhances the operational safety of virtually coupled monorail formations. However, if the safety distance is improperly configured, the effectiveness of the collision avoidance system is greatly diminished, rendering it incapable of ensuring safety. The appropriate safety distance primarily depends on the braking distance of the MRT vehicles, which includes both normal braking [36] and emergency braking [37]. When the inter-vehicle spacing decreases to the safety threshold and the MRT vehicles must rapidly reduce their speed, the collision avoidance system can directly activate emergency braking. Therefore, the braking distance used to calculate the safety distance should be based on the vehicle parameters under emergency braking conditions, with the selection of the longest possible braking distance (the distance over which an MRT vehicle is brought to a full halt when braking from its peak operating speed). However, the safety distance should not be determined solely by the maximum braking distance. Due to potential external environmental factors and the possibility of communication delays within the monorail system, emergency braking may still be affected. As a result, the safety distance should consist of both the maximum braking distance and an additional safety buffer margin. This configuration is illustrated in Fig 20, where $d_{b}$ denotes the maximum emergency braking distance and $d_{m}$ represents the safety buffer margin.

As shown in the figure, the safety distance for the collision avoidance system of virtually coupled MRT vehicles is defined as:


$$d_{s}=d_{b}+d_{m}$$ (23)

The correlation between rail vehicle speed and braking distance is described by:


$$v=\frac{\Delta d_{s}}{\Delta t}$$ (24)

Where Δt is the time interval, and $\Delta d_{s}$ denotes the change in safety distance within the time interval. Under the assumption of no curved tracks or gradients, the average deceleration of the MRT vehicle during emergency braking is given by:


$$a_{s}=\frac{v\cdot a}{v+2\cdot a\cdot t_{d}}$$ (25)

Where $t_{d}$ represents the equivalent response time of the MRT vehicle during emergency braking, during which the vehicle covers a coasting distance. In this simulation experiment, it is set to 1s. Accordingly, the maximum braking distance of the MRT vehicle is given by:


$$d_{b}=\frac{v^{\text{2}}}{2\cdot a_{s}}$$ (26)

**Fig 21:**
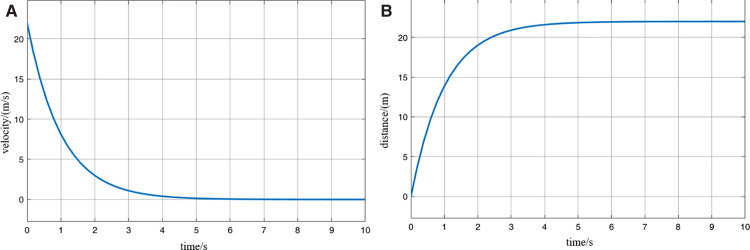
Curve of braking and parking speed and distance of MRT vehicles, (A) braking stop speed curve, (B) braking stop distance curve.

The longest braking distance of the MRT vehicle is determined through simulation. The highest operating speed of the MRT vehicle is typically 80 km/h. Under full passenger load conditions, the vehicle applies maximum braking force starting from the initial moment. The resulting speed and distance curves are shown in Fig 21. From the figure, it can be observed that during emergency braking until the vehicle speed reaches 0, the MRT vehicle travels a distance of 23 m. Therefore, the maximum braking distance $d_{b}$ of the MRT vehicle is 23 m. This value is derived under standard conditions (dry track and new brake pads). Under extreme conditions—for example, with an adhesion coefficient of 0.3—the required safety distance should be increased. The present study, therefore, focuses on standard operating conditions.

The determination of the safety buffer distance for virtually coupled MRT vehicles requires comprehensive consideration of multiple factors, including track characteristics, weather conditions, track slipperiness, vehicle weight, and internal communication delays within the monorail system. In [38], after accounting for these factors, the safety distance in a moving block system is set to be 3–4 times the safety buffer distance. Accordingly, based on the maximum braking distance of the MRT vehicle, the safety buffer distance is determined to be 5 m. Combining the above analysis for selecting the safety distance, the final safety distance $d_{s}$ for the MRT vehicle is established as 28 m.

To verify the effectiveness of the proposed collision avoidance strategy and control algorithm for virtually coupled MRT vehicles, a virtual formation consisting of three MRT vehicles is selected as the simulation subject. Based on the MRT vehicle safety distance $d_{s}$, the VC formation spacing $d_{f}$ is set to 30 m. Using relevant parameters of typical driver-cab vehicles on Chongqing Rail Transit Line 3, a simulation experiment of acceleration and collision avoidance during station departure is established. To approximate real operational conditions, each MRT vehicle in operation is assigned an initial position and initial speed, and the vehicles operate under three loading conditions: empty, full load, and overloaded. The empty vehicle mass is $M_{\text{1}}=28600kg$, the full load mass is $M_{\text{2}}=41290kg$, and the overloaded vehicle mass is $M_{\text{3}}=53920kg$. It is assumed that during gradual acceleration departure from the station, communication faults such as excessive delays in vehicle-to-ground and V2V communication caused inconsistent acceleration among the vehicles within the virtual formation, resulting in speed differences. At a certain initial moment, the positions and initial speeds of the three monorail vehicles are given in Table 5.

**Table 5:** Initial Conditions for Accelerating Exit Collision Avoidance Scenarios.

Application scenario	Accelerating Exit Collision Avoidance	
**MRT vehicles**	**Initial velocity (km/h)**	**Distance to the preceding vehicle (m)**
**Leading vehicle**	20	0
**Following vehicle 1**	30	30
**Following vehicle 2**	20	30

**Fig 22:**
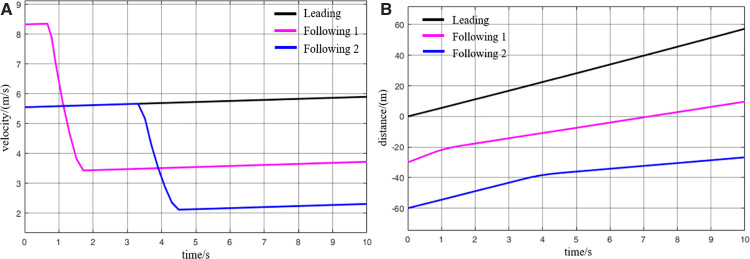
MRT vehicles' acceleration exit collision avoidance process (empty vehicle status), (A) velocity curve, (B) distance curve.

**Fig 23:**
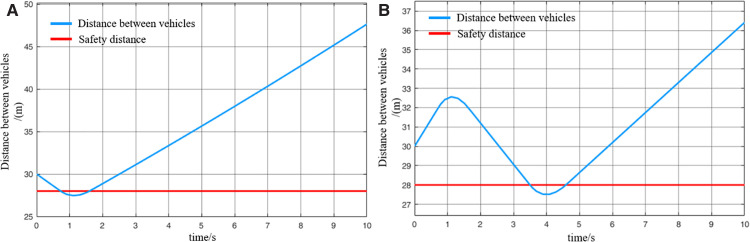
Change in distance between trains during the collision avoidance process (empty vehicle status), (A) leading vehicle-following vehicle 1 spacing, (B) following vehicle 1-following vehicle 2 spacing.

When the virtually coupled MRT vehicles operate in the empty-load condition and accelerate to depart from the station, the collision avoidance decision-making process is illustrated in Fig 22. Fig 23 shows the variation curve of inter-vehicle spacing during the execution of the collision avoidance process. As shown in the figure, due to control and communication faults, the speed of the following vehicle 1 abnormally increases, resulting in a continuous decrease in the distance between it and the leading vehicle. At *t* = 0.8 s, the inter-vehicle spacing decreases to the predefined safety distance, triggering the activation of the MRT vehicle’s collision avoidance system, which then initiates emergency braking. The emergency braking process for following vehicle 1 lasts for 0.8 s, during which its speed decreases to 3.5 m/s, and the distance from the leading vehicle returns to beyond the safety threshold. The collision avoidance system is subsequently deactivated. Once the emergency braking is triggered, the following vehicle 1 automatically disengages from the VC with the leading vehicle. Therefore, after *t* = 1.6 s, in the absence of new instructions from the control center (cloud platform), following vehicle 1 continues moving forward at its current speed. Due to the deceleration of the following vehicle 1, the motion state of the following vehicle 2 is inevitably affected. At *t* = 3.4 s, the spacing between following vehicle 2 and following vehicle 1 decreases to the safety distance, which then triggers the activation of the collision avoidance system on following vehicle 2. Emergency braking is initiated and continues until the inter-vehicle spacing returns to above the safety threshold, at which point the system is deactivated. Through this process, the virtually coupled monorail formation successfully performs collision avoidance, ensuring the operational safety of the vehicles under unexpected conditions.

It is worth noting that once the MRT vehicle’s collision system is deactivated, the vehicle will continue to drive in the acceleration state before emergency braking, unless there is a new control signal issued by the control center (cloud platform). Since the entire collision avoidance system does not rely on V2V or vehicle-to-ground communication, and is solely triggered by the safety distance while evaluating only the vehicle’s own operating state. Consequently, the lead vehicle remains unaffected during this process. It continues to accelerate according to its original command before the fault occurrence. Numerical simulation results demonstrate that the proposed proactive collision avoidance strategy and model predictive control algorithm can effectively ensure the safety of MRT vehicles during acceleration departure, thereby validating the effectiveness of the proposed control approach. While the collision avoidance simulation for virtually coupled MRT vehicles under empty-load acceleration successfully achieves active safety control, real-world scenarios often involve vehicles in different loading conditions. Under such conditions, the mass of individual MRT vehicles varies, and vehicle mass has a significant impact on emergency braking performance. Therefore, further simulations are required to evaluate the collision avoidance process under varying passenger load conditions. Compared to the collision avoidance process under the empty-load condition, the decision-making procedure during acceleration departure with passenger loading remains largely unchanged.

**Fig 24:**
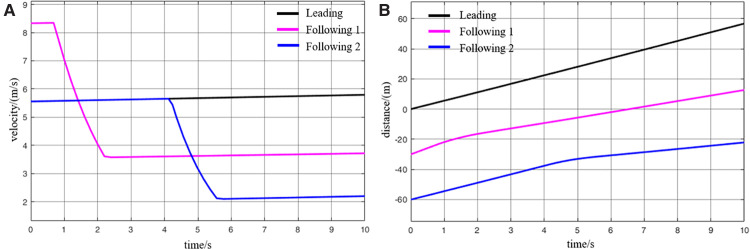
MRT vehicles' acceleration and exit collision avoidance process (passenger state), (A) velocity curve, (B) distance curve.

**Fig 25:**
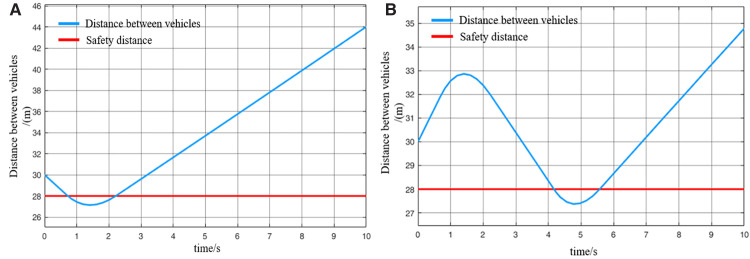
Change in distance between trains during the collision avoidance process (passenger state), (A) leading vehicle-following vehicle 1 spacing, (B) following vehicle 1-following vehicle 2 spacing.

When MRT vehicles are stationed for passenger boarding, the distribution of passengers along the platform typically follows an approximately normal distribution. As a result, the lead and rear vehicles tend to carry fewer passengers compared to the middle vehicle. Thus, it can be assumed that during acceleration departure of virtually coupled MRT vehicles, the leading vehicle and following vehicle 2 operate under full-load conditions, while following vehicle 1 operates under an overloaded condition. The collision avoidance decision-making process is illustrated in Fig 24, and the variation of inter-vehicle spacing during the avoidance process is shown in Fig 25. However, a key difference lies in the prolonged emergency braking phase. Due to the increased inertia of the overloaded following vehicle 1, its emergency braking process lasts for 1.5 s—approximately twice as long as in the empty-load condition. As a result of this extended braking duration, the activation of the collision avoidance system for following vehicle 2 is also delayed by approximately 0.8 s, and its emergency braking duration is similarly extended. Overall, a comparison of the two scenarios indicates that the collision risk during acceleration departure under loaded conditions is slightly higher than in the empty-load condition. Nevertheless, the numerical simulation results confirm that the proposed proactive collision avoidance strategy and model predictive control algorithm can effectively ensure the operational safety of virtually coupled MRT vehicles across different loading conditions.

The above experimental results indicate that during acceleration departure, the probability of inter-vehicle collision in virtually coupled monorail formations remains low, regardless of whether the vehicles operate in an empty-load or passenger-loaded condition. This is primarily because, during the acceleration phase, the leading vehicle is continuously speeding up, effectively “escaping” from the following vehicles. As a result, even a brief deceleration by the following vehicles is sufficient to maintain a safe inter-vehicle distance and ensure operational safety. In contrast, the scenario of deceleration for station arrival presents a higher risk. In this case, the leading vehicle is decelerating, which resembles “waiting” for the following vehicles to catch up. This significantly increases the likelihood of inter-vehicle collisions and makes collision avoidance for the following vehicles more challenging. To validate the proposed collision avoidance strategy under this scenario, a simulation is conducted for virtually coupled MRT vehicles decelerating for station arrival. It is assumed that a three-vehicle virtual formation begins to decelerate at a certain distance from the station in preparation for stopping. Due to faults such as excessive delays in vehicle-to-ground or inter-vehicle communication, the vehicles within the formation decelerate inconsistently, resulting in speed differences. At a given initial time, the positions and speeds of the three monorail vehicles are shown in Table 6.

**Table 6:** Initial conditions for deceleration entry Collision Avoidance Scenarios.

Application scenarios	Deceleration Entry Collision Avoidance		
**MRT vehicles**	**Initial velocity (km/h)**	**Distance to the station (m)**	**Distance to the preceding vehicle (m)**
**Leading vehicle**	20	100	/
**Following vehicle 1**	30	130	30
**Following vehicle 2**	36	160	30

**Fig 26:**
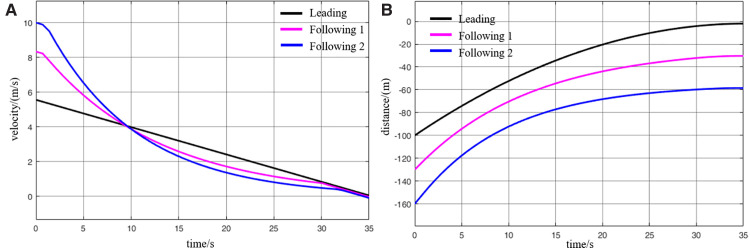
MRT vehicles deceleration enter the collision avoidance process (empty vehicle status), (A) velocity curve, (B) distance curve.

**Fig 27:**
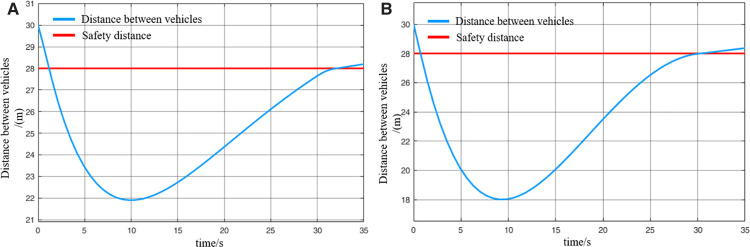
Change in distance between trains during the collision avoidance process (empty vehicle status), (A) leading vehicle-following vehicle 1 spacing, (B) following vehicle 1-following vehicle 2 spacing.

When virtually coupled MRT vehicles operate in the empty-load condition and decelerate for station arrival, their collision avoidance decision-making process is illustrated in Fig 26, and the variation in inter-vehicle spacing during the avoidance process is shown in Fig 27. As observed from the figures, due to control and communication faults, the vehicles within the formation decelerate inconsistently, resulting in the following vehicle 1 having a higher initial speed than the leading vehicle. Since the leading vehicle is braking to decelerate for station entry, the distance between it and the following vehicle 1 rapidly decreases to the safety threshold, creating a high risk of collision. Upon detecting that the inter-vehicle spacing has reached the safety distance, the following vehicle 1 activates the emergency collision avoidance system and begins emergency braking. At *t* = 9.5 s, its speed finally decreases to match that of the leading vehicle, at which point the inter-vehicle spacing reaches its minimum of 18 m. Following vehicle 1 then continues emergency braking until the distance from the leading vehicle exceeds the safety threshold again, at which point the collision avoidance system is deactivated. The entire braking process lasts for 29.4 s, during which the vehicle slows down to 0.8 m/s. Thereafter, the vehicle continues to move under its previous deceleration state and proceeds to stop at the station. The emergency deceleration of the following vehicle 1 inevitably affects the motion state of the following vehicle 2. Almost simultaneously with the gap between following vehicle 1 and the leading vehicle reaching the safety threshold, the spacing between following vehicle 2 and following vehicle 1 also decreases to the safety limit. This triggers the activation of the emergency collision avoidance system on the following vehicle 2, initiating its braking process. The emergency braking of the following vehicle 2 lasts even longer, up to 32s, after which the vehicle slows to 0.4 m/s and continues decelerating until it arrives at the station. Through this process, the virtually coupled monorail formation completes collision avoidance during deceleration for station arrival.

**Fig 28:**
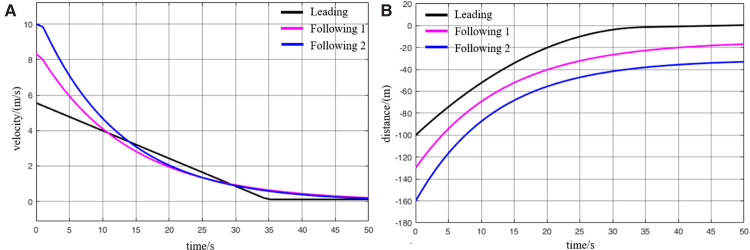
MRT vehicles' acceleration enter collision avoidance process (passenger state), (A) velocity curve, (B) distance curve.

**Fig 29:**
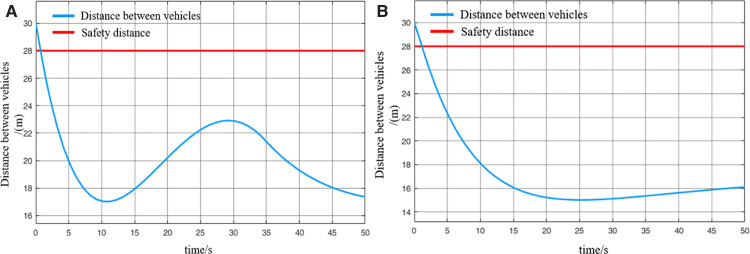
Change in distance between trains during the collision avoidance process (passenger state), (A) leading vehicle-following vehicle 1 spacing, (B) following vehicle 1-following vehicle 2 spacing.

As with the acceleration departure collision scenario, deceleration for station entry scenarios for virtually coupled MRT vehicles also requires collision avoidance simulations under different loading conditions. In this case, it is assumed that during deceleration and station approach, the leading vehicle and following vehicle 2 operate under fully loaded conditions, while the following vehicle 1 is in an overloaded state. The collision avoidance decision-making process is illustrated in Fig 28, and the inter-vehicle spacing variation during the execution process is shown in Fig 29. From the figures, it can be observed that, unlike the empty-load deceleration scenario, the collision avoidance process under loaded conditions is significantly different. Both following vehicle 1 and following vehicle 2 remain in emergency braking mode throughout the station approach. Analyzing the following vehicle 1 first, its avoidance process can be divided into two stages, separated at *t* = 35 s, when the leading vehicle completes deceleration and comes to a stop. In the first stage, the emergency collision-avoidance mode is triggered once the following vehicle 1 and the leading vehicle gap reaches the safety limit. The following vehicle 1 begins braking and reduces its speed to match that of the leading vehicle at *t* = 10 s, at which point the minimum inter-vehicle spacing reaches 18 m. After that, although the vehicle remains in an emergency braking state and its speed becomes lower than that of the leading vehicle, the inter-vehicle spacing begins to increase gradually. However, even after the leading vehicle comes to a complete stop, the spacing never exceeds the safety threshold. In the second stage, since the leading vehicle is stationary while the following vehicle 1 continues braking, the speed of following vehicle 1 continues to decrease, causing the inter-vehicle distance to shrink again, until it eventually comes to a safe stop. The avoidance process for following vehicle 2 can also be divided into two stages. In the first stage, similar to following vehicle 1, the vehicle performs emergency braking as the inter-vehicle spacing decreases. In the second stage, after *t* = 20 s, following vehicle 2 reduces its speed to approximately match that of following vehicle 1, and the spacing between the two vehicles stabilizes. It then continues braking until it comes to a complete stop at a safe distance. Thus, the entire virtually coupled monorail formation completes collision avoidance during deceleration and station approach.

By comparing the collision avoidance decision-making processes during station departure and station approach, it can be observed that the risk of collision is significantly higher during deceleration for station entry than during acceleration for departure, particularly under peak passenger flow conditions when MRT vehicles are fully loaded. Therefore, special attention should be given to safety protection measures in this scenario during system safety design. Numerical simulation results demonstrate that even under high collision risk during deceleration and station approach, the proposed proactive collision avoidance strategy and model predictive control algorithm for virtually coupled MRT vehicles can still effectively ensure operational safety.

The collision avoidance scenarios during acceleration for station departure and deceleration for station approach have already covered the majority of high-risk situations involving urban rail transit vehicles. However, it is also necessary to consider the possibility of sudden obstacles appearing on the track while virtually coupled MRT vehicles are in motion, potentially resulting in collisions. This article considers accidents in emergencies, where obstacles are generated due to accident reasons when the VC MRT vehicles arrive. At this time, the time delay of the monitoring device cannot be avoided, and only by relying on the active collision avoidance technology of the MRT vehicles themselves can collision accidents be avoided. Unlike the station departure and arrival scenarios, virtually coupled MRT vehicles operating on open track sections are generally in a uniform-speed state. When an obstacle appears ahead, the collision avoidance procedure must be initiated by the lead vehicle. In this scenario, it is assumed that a virtually coupled train of three monorail vehicles is traveling at a constant speed on the track. An obstacle suddenly appears 50 m ahead of the lead vehicle due to an external accident. The vehicles under study are based on those of Chongqing Rail Transit Line 3, whose typical operating speed on open tracks ranges from 20 to 35 km/h. In this simulation, a representative speed of 25 km/h is selected. At a specific initial time, the position and initial speed of each of the three MRT vehicles are shown in Table 7.

**Table 7:** Initial Conditions for Obstacle Avoidance Scenarios.

Application scenarios	Obstacle avoidance		
**MRT vehicles**	**Initial velocity(km/h)**	**Distance to obstacle walls(m)**	**Distance to the preceding vehicle(m)**
**Leading vehicle**	25	50	/
**Following vehicle 1**	25	80	30
**Following vehicle 2**	25	110	30

**Fig 30:**
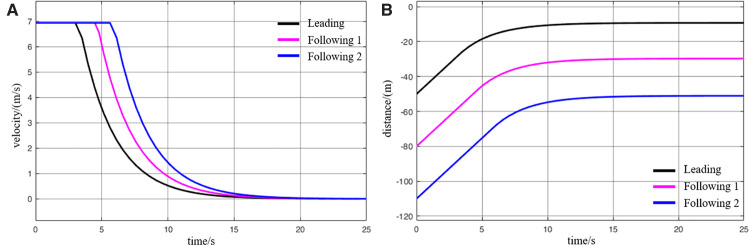
The process of avoiding collisions of MRT vehicles encountering obstacles (empty vehicle state), (A) velocity curve, (B) distance curve.

**Fig 31:**
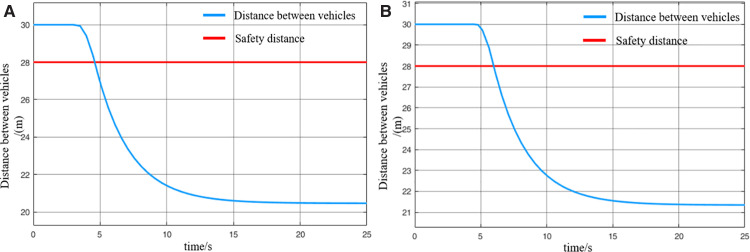
Changes in the distance between trains during collision avoidance (empty vehicle state), (A) leading vehicle-following vehicle 1 spacing, (B) following vehicle 1-following vehicle 2 spacing.

When the virtually coupled MRT vehicles are operating in an empty (unloaded) state while encountering an obstacle on the track, their collision avoidance decision-making process is illustrated in Fig 30, while Fig 31 shows the variation in inter-vehicle distances during the avoidance process. As can be seen from the figures, the emergency braking patterns of the three MRT vehicles are consistent, differing only in their sequence of activation. The leading vehicle initiates emergency braking immediately upon detecting that the distance to the obstacle ahead has decreased to the predefined safety threshold. Meanwhile, follower vehicles 1 and 2 continue to travel at constant speed. At *t* = 4.5 s, the inter-vehicle distance between the leading vehicle and following vehicle 1 reaches the safety threshold, triggering following vehicle 1 to begin emergency braking. Similarly, at *t* = 5.5 s, following vehicle 2 detects that its distance to following vehicle 1 has reached the safety threshold, prompting it to initiate emergency braking as well. Subsequently, all three MRT vehicles continue moving forward in an emergency braking state, gradually approaching a dynamic equilibrium until coming to a complete stop. Thus, the virtually coupled monorail formation completes the obstacle avoidance maneuver. This process is relatively straightforward, as it does not involve variations in vehicle speed during normal operation before the emergency response.

**Fig 32:**
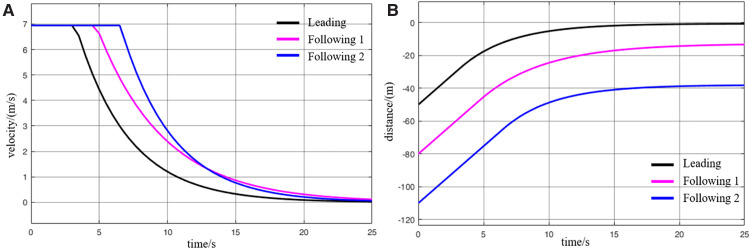
The process of avoiding collisions of MRT vehicles encountering obstacles (passenger state), (A) velocity curve, (B) distance curve.

**Fig 33:**
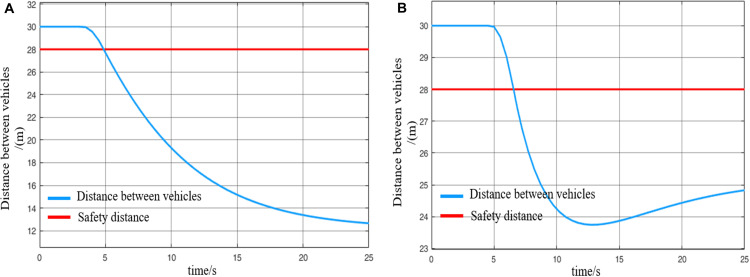
Changes in the distance between trains during collision avoidance (passenger state), (A) leading vehicle-following vehicle 1 spacing, (B) following vehicle 1-following vehicle 2 spacing.

It is also necessary to conduct simulation verification of obstacle avoidance in scenarios where virtually coupled MRT vehicles operate under different passenger load conditions. Suppose that when the virtually coupled monorail formation encounters an obstacle while operating on the track, the leading vehicle and following vehicle 2 are in a fully loaded state, while following vehicle 1 is in an overloaded state. The collision avoidance decision-making process is shown in Fig 32, and Fig 33 illustrates the variation in inter-vehicle distances during the avoidance process. From the figures, the lead vehicle and follower vehicle 2 exhibit nearly the same emergency-braking profile because they share the same load setting. As before, the leading vehicle is the first to detect that the distance to the obstacle ahead has reached the safety threshold and initiates the emergency collision avoidance system. Following vehicles 1 and 2 continue to travel at a constant speed. At *t* = 4.5 s, the distance between the leading vehicle and following vehicle 1 reaches the safety threshold, prompting following vehicle 1 to begin emergency braking. At *t* = 6.5 s, the gap between following vehicle 1 and following vehicle 2 also reaches the safety threshold, triggering following vehicle 2 to initiate emergency braking. Due to its overloaded state, the following vehicle 1 exhibits weaker braking performance compared to the leading vehicle and the following vehicle 2. At *t* = 12.5 s, following vehicle 2’s speed decreases to match that of following vehicle 1, after which it continues to decelerate at a slightly lower speed. As a result, the inter-vehicle distance between following vehicles 2 and 1 initially decreases and then increases. Ultimately, all three MRT vehicles successfully perform emergency braking before reaching the obstacle, completing the collision avoidance maneuver.

## 5. Conclusion

This paper focuses on the active collision avoidance control technology for intelligent connected monorail transit systems under a VC framework. For the first time, a control model for both leading and following vehicles in a virtually coupled environment is developed. By integrating an MPC algorithm, the system enables dynamic formation and coordination of virtual trainsets across various operational scenarios. Based on this framework, typical operating conditions are further analyzed to develop effective collision avoidance strategies for virtually coupled vehicles. Simulation experiments, using Chongqing Rail Transit Line 3 as a case study, validate the effectiveness of the proposed model and algorithm. The results demonstrate that the system can achieve efficient formation scheduling across multiple coupling scenarios while ensuring operational safety in the face of unexpected events.

However, to simplify the modeling process during simulation, the MRT vehicle is abstracted as a point mass, which inevitably overlooks many practical influencing factors. The wheel system structure plays a critical role in the operational dynamics of monorail vehicles and should therefore be fully considered in simulation modeling. However, the current model does not yet incorporate this aspect. Within this technical scope, the present study primarily focuses on the modeling and control-algorithm design for coordinated train operations under virtual coupling, rather than managerial aspects such as institutional arrangements, operational organization, human resources, or cost management.

Future research will focus on introducing more complex and refined vehicle structural models to enhance the physical realism and engineering applicability of the simulations. This will enable more accurate evaluations of system performance and operational safety. In addition, future work will incorporate operational data and implementation constraints to further examine the managerial implications of deploying virtual coupling, including operational organization and management decision-making, as well as cost-benefit analysis. These findings highlight the significant potential and practical value of VC technology and proactive collision avoidance strategies in urban rail transit systems, providing a solid theoretical foundation and technical support for future engineering applications of intelligent connected transit systems.
